# Effectiveness of different types of exercise therapy in improving post-stroke depression: a systematic review and network meta-analysis

**DOI:** 10.3389/fmed.2026.1828873

**Published:** 2026-05-01

**Authors:** Wenna Li, Lingxu Li, Sainan Zhao, Chunmeng Wang, Yanlong Jin, Guangcheng Ji

**Affiliations:** 1 Department of Neurology, Changchun University of Traditional Chinese Medicine, Changchun, Jilin, China; 2 Department of Neurology, The Third Clinical Hospital Affiliated to Changchun University of Traditional Chinese Medicine, Changchun, Jilin, China

**Keywords:** exercise therapy, network meta-analysis, post-stroke depression, randomized controlled trial, systematic review

## Abstract

**Objective:**

To systematically evaluate the effects of exercise therapies such as aerobic exercise (CAE), Tai Chi (TC), Baduanjin (BDJ), resistance training (RE), Wuqinxi (WQX), yoga (YG), and rehabilitation training (RT) on depressive symptoms, daily living abilities, motor function, and balance function in patients with post-stroke depression (PSD). A network meta-analysis method was employed to compare the relative efficacy of various interventions, providing high-quality evidence-based medical data for clinical selection of optimal intervention strategies.

**Methods:**

We conducted systematic searches in China National Knowledge Infrastructure, Wanfang Data, VIP Information, Chinese Biomedical Literature Database, PubMed, Embase, Cochrane Library, and Web of Science, with the search period concluding on April 5, 2026. Randomized controlled trials (RCTs) evaluating exercise therapy interventions for PSD were included. Two investigators independently performed screening, data extraction, and RoB 2.0 bias risk assessment. A network meta-analysis was performed using Stata 17.0 under the frequency framework, with continuous outcomes expressed as mean difference (MD) and 95% confidence interval (CI), and ranked by SUCRA.

**Results:**

A total of 33 RCTs (*n* = 2,339) were included, comparing seven types of interventions: CAE, TC, BDJ, RE, WQX, YG, and RT. The network meta-analysis revealed that compared with the control group, RT [MD = −8.45, 95% CI (−11.59, −5.31)], WQX [MD = −5.55, 95% CI (−11.00, −0.10)], BDJ [MD = −3.41, 95% CI (−6.24, −0.58)], and TC [MD = −3.02, 95% CI (−5.32, −0.71)] significantly reduced the Hamilton Depression Rating Scale (HAMD) scores; CAE [MD = −12.48, 95% CI (−17.79, −7.17)] significantly decreased Self-Rating Depression Scale (SDS) scores; RT[MD = 9.47, 95% CI (2.39, 16.55)] demonstrated the largest effect size in improving the Barthel Index (BI) scores; RE (SUCRA = 96.7%) was the most effective intervention for improving the Fugl-Meyer Assessment (FMA) scores; BDJ [MD = 8.20, 95% CI (2.73, 13.67)] significantly enhanced the Berg Balance Scale (BBS) levels. HAMD and SDS were used to assess patients’ depressive status, BI to evaluate daily living abilities, FMA to assess motor function, and BBS to evaluate balance function.

**Conclusion:**

Exercise therapy can serve as an effective adjunctive intervention in the comprehensive management of PSD, demonstrating potential benefits in alleviating depressive symptoms and improving certain functional outcomes. The efficacy of different exercise modalities varies across outcome dimensions: RT shows superior performance in improving HAMD scores and BI, CAE exhibits greater advantages in enhancing SDS scores, RE ranks highest in promoting FMA improvement, and BDJ demonstrates optimal efficacy in improving BBS scores. Given the overall limited methodological quality of included studies, small sample sizes for some outcomes, relatively sparse evidence networks, and short follow-up durations in most studies, these findings require further validation through high-quality, large-sample, long-term randomized controlled trials.

**Systematic review registration:**

CRD420261333356.

## 1 Introduction

Stroke is one of the leading causes of death and disability worldwide. The Global Burden of Disease (GBD) study indicates that the number of stroke events and stroke-related deaths globally showed an overall upward trend from 1990 to 2021, with stroke remaining a significant cause of long-term functional impairment and disease burden (1, 2). PSD is one of the most common neuropsychiatric complications following stroke. Systematic reviews indicate that the prevalence of depression or depressive symptoms at any time point after stroke is approximately one-third, and PSD is closely associated with poor functional recovery, decreased quality of life, and increased risk of mortality (3–5).

Non-pharmacological treatments, particularly exercise therapy, have been widely applied in stroke rehabilitation practice. Previous studies have demonstrated that exercise intervention not only helps improve balance function, motor ability, and activities of daily living in stroke patients, but may also exert positive effects on cognitive function and overall quality of life (6–9). However, current evidence on exercise-induced improvement of PSD primarily focuses on single exercise modalities or traditional pairwise comparisons. While suggesting that physical activity may generally be beneficial, the relative efficacy and prioritization of different exercise therapies in PSD-related outcomes remain unclear. Network meta-analysis enables simultaneous comparison and ranking of multiple interventions, thereby providing more comprehensive evidence-based support for clinical selection of optimal exercise intervention regimens (10, 11).

Based on the aforementioned background, this study employs a network meta-analysis approach to systematically evaluate the impact of exercise therapy on patients with PSD. The study aims to compare the relative efficacy of different exercise therapies and identify optimal intervention strategies, thereby providing high-quality evidence-based medicine for clinical practice and offering novel approaches for comprehensive rehabilitation treatment of PSD.

## 2 Materials and methods

### 2.1 Study design and registration

This study was reported in strict compliance with the PRISMA 2020 guidelines and its Extended Statement for Network Meta-Analyses (PRISMA-NMA). Registration number (CRD420261333356).

### 2.2 Inclusion and exclusion criteria

Develop standards based on the PICOS principles.

Inclusion criteria:

(1) Participants: Adults (≥18 years) with depression or depressive symptoms after stroke; stroke diagnosis based on recognized clinical or imaging criteria (e.g., AHA/ASA definitions) (12), and depression assessed using standardized scales or DSM/ICD diagnostic criteria.
(2) Interventions: Tai Chi, Baduanjin, Wuqinxi, yoga, aerobic exercise, resistance training, and rehabilitation training.
(3) Comparators: Usual care, conventional treatment, or routine rehabilitation.
(4) Outcomes: HAMD, SDS, BI, FMA, and BBS.
(5) Study design: Randomized controlled trials.

Exclusion criteria:

(1) Non-randomized controlled studies, reviews, case reports, and conference abstracts.
(2) Duplicate publications.
(3) Inability to obtain full text or key data.
(4) Interventions or outcomes not meeting the study requirements.

### 2.3 Literature retrieval strategy

Two researchers independently searched the China National Knowledge Infrastructure, Wanfang Data, VIP Information, Chinese Biomedical Literature Database, PubMed, Embase, Cochrane Library, and Web of Science, with the search period spanning from the database establishment to April 5, 2026. The search employed a combination of subject terms and free words, utilizing Boolean logic terms (“AND,” “OR,” “NOT”) to precisely filter relevant literature. Additionally, comprehensive searches were conducted for previously published reviews and other meta-analyses on related topics to identify undiscovered studies. Supplementary literature was further supplemented by reviewing references cited in the retrieved articles.

### 2.4 Literature screening and data extraction

Two researchers (Li Wenna and Li Lingxu) independently conducted literature screening and data extraction: initial screening was performed by reviewing abstracts and titles, followed by full-text verification with documentation of exclusion criteria. Extracted information included author and year, study design, sample size, baseline characteristics, intervention measures, control measures, outcome scales and measurement time points, as well as follow-up and adverse events. Disagreements were first resolved through discussion; if consensus could not be reached, a third researcher (Zhao Sainan) made the final determination. Missing data were supplemented by contacting the authors whenever possible.

### 2.5 Bias risk assessment

To ensure the reliability and consistency of the assessment results, this study employed the RoB 2.0 tool to conduct bias risk assessments for all included randomized controlled trials (RCTs) (11). The RoB 2.0 tool introduces specific signal questions, which clarify the bias assessment process, reduce the impact of subjective bias, and enhance the accuracy and consistency of evaluations. The tool comprises five primary domains: bias in randomization processes, intervention bias, measurement bias, missing data bias, and selection reporting bias. Each domain is assessed based on responses to signal questions, with bias risks categorized as low, high, or several concerns. The RoB 2.0 tool incorporates specific signal questions for each domain, facilitating the identification of bias sources and further improving assessment transparency. We utilized these signal questions for detailed analysis in each study and labeled the bias risks for individual studies. The overall study-level bias risk was determined based on domain-specific assessment results. Quality evaluations were independently conducted by two researchers (Li Wenna, Li Lingxu), with disagreements resolved by a third researcher (Zhao Sainan).

### 2.6 Statistical analysis

Since not all included studies reported all prespecified outcomes, the number of network meta-analyses for different outcomes varied. This study employed the network and mvmeta software packages in Stata 17.0 to conduct network meta-analyses within a frequency mathematical framework. Only outcomes for which sufficient quantitative data were reported for analysis were included in the corresponding network meta-analysis. All included outcomes were continuous variables, including: HAMD and SDS for depression symptom assessment, BI for daily living activities assessment, FMA for motor function assessment, and BBS for balance function assessment.

For each included study and outcome measure, sample sizes, mean values after intervention, and standard deviations for all study groups were extracted whenever possible. When studies reported both baseline and post-intervention values, endpoint data at the end of intervention were prioritized for primary analysis to ensure consistency in effect size estimation across studies. Since the scales used within each outcome network were consistent, effect sizes were expressed as mean difference (MD) and its 95% confidence interval (CI).

First, a network relationship diagram is drawn to illustrate the comparative relationships among different interventions and the existing evidence structure. Nodes in the diagram represent various interventions, with node size proportional to the total sample size receiving the intervention. The thickness of connecting lines reflects the number of direct comparative studies between two interventions.

Considering the anticipated clinical and methodological heterogeneity in study inclusion regarding exercise intervention types, intervention intensity, treatment duration, participant characteristics, and conventional therapeutic measures, this study employed a random effects model for network meta-analysis. In this study, we hypothesized that all exercise therapies were comparable and that patient background characteristics would not significantly influence the comparison results between interventions. To test this hypothesis, meta-regression analysis was conducted, with results demonstrating no significant impact. For consistency assessment, we considered using network meta-analysis and applied node splitting when necessary. However, since the network graphs in this study did not form closed-loop structures, node splitting was unsuitable for consistency evaluation.

Forest plots were used to display the effect sizes and 95% confidence intervals of each intervention relative to the control group. The cumulative area under the rank-order curve (SUCRA) was employed to rank the efficacy probabilities of different interventions, with values ranging from 0 to 100%, where higher values indicate greater likelihood of the intervention being superior. It should be noted that SUCRA reflects ranking probabilities rather than absolute efficacy magnitude; therefore, result interpretation primarily relies on effect sizes, 95% confidence intervals, and evidence precision, with ranking results serving only as supplementary information. Comparison-adjusted funnel plots were utilized to explore the possibility of small-sample effects or publication bias.

For missing or incompletely reported outcome data, we first reviewed the original text, tables, figures, and supplementary materials to extract available additional information, and contacted the original study authors for clarification when necessary. If key quantitative data (e.g., sample size, mean, or standard deviation) were unavailable or could not be reliably converted, the study was excluded from quantitative synthesis of corresponding outcomes but retained in qualitative descriptions where appropriate. For issues of lost-to-follow-up or incomplete outcome data, if the original study had reported corresponding handling methods, we extracted data according to the original text; bias risks associated with missing outcome data were reflected in the RoB 2.0 assessment.

## 3 Results

### 3.1 Literature retrieval results

**Figure 1:**
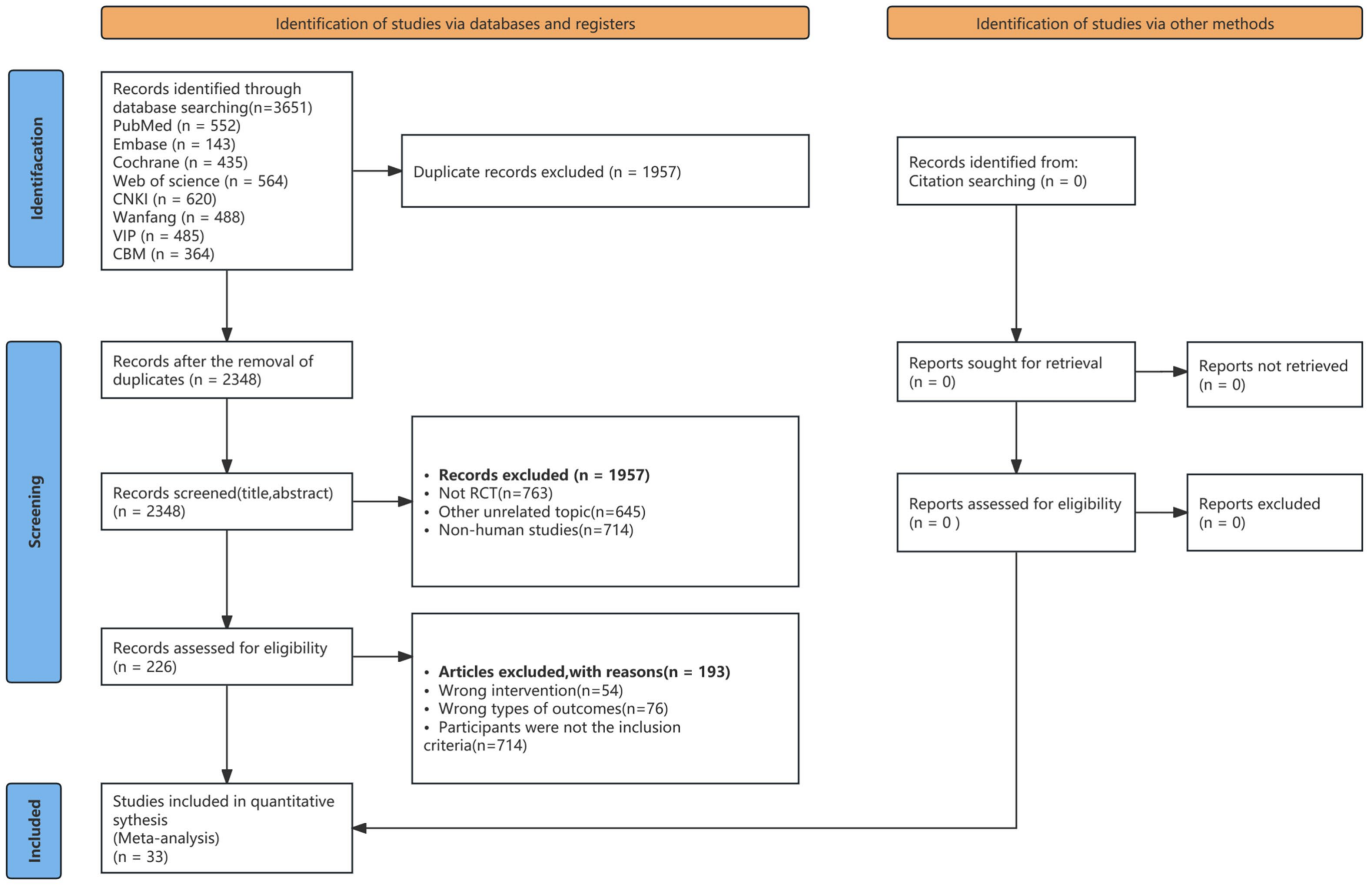
Research selection flowchart. Literature screening flowchart “The flowchart illustrates the screening process of studies included in this systematic review and network meta-analysis, including database searches, removal of duplicates, initial screening, full-text assessment, and the final inclusion of 33 randomized controlled trials (RCTs)”.

Figure 1 illustrates the literature screening process. Initially, a total of 3,240 studies were retrieved from the database. Subsequently, duplicate articles and studies that did not meet inclusion criteria were excluded. After comprehensive text review, 33 articles were deemed eligible for inclusion in the meta-analysis. Among these, 19 were in Chinese and 14 in English, involving a total of 2,339 participants. The publication years of these studies ranged from 2006 to 2025. For detailed descriptions of the search terms used, please refer to Supplementary Table S1.

### 3.2 Basic characteristics of enrolled studies

A total of 33 studies were included, all of which were randomized controlled trials. These studies evaluated conventional treatments versus seven exercise interventions, including Continuous Aerobic Exercise (CAE); Tai Chi (TC); Resistance Exercise (RE); Baduanjin Exercise (BDJ); Wuqinxi Exercise (WQX); Yoga (YG); and Rehabilitation Training (RT). The overview table of literature included in the study is provided in Supplementary Table S2, and the basic characteristics of the included literature are presented in Supplementary Table S3.

### 3.3 Bias risk assessment for study inclusion

This study employed the Cochrane RoB 2.0 tool to conduct a bias risk assessment on the 33 randomized controlled trials (RCTs) included. The RoB 2.0 tool evaluated potential bias risks across five primary aspects: randomization process, intervention delivery, measurement methods, missing data, and selective reporting. Each aspect was analyzed in detail based on specific signal questions to determine the bias risk level of each study in these areas: low risk, high risk, or multiple concerns.

**Figure 2:**
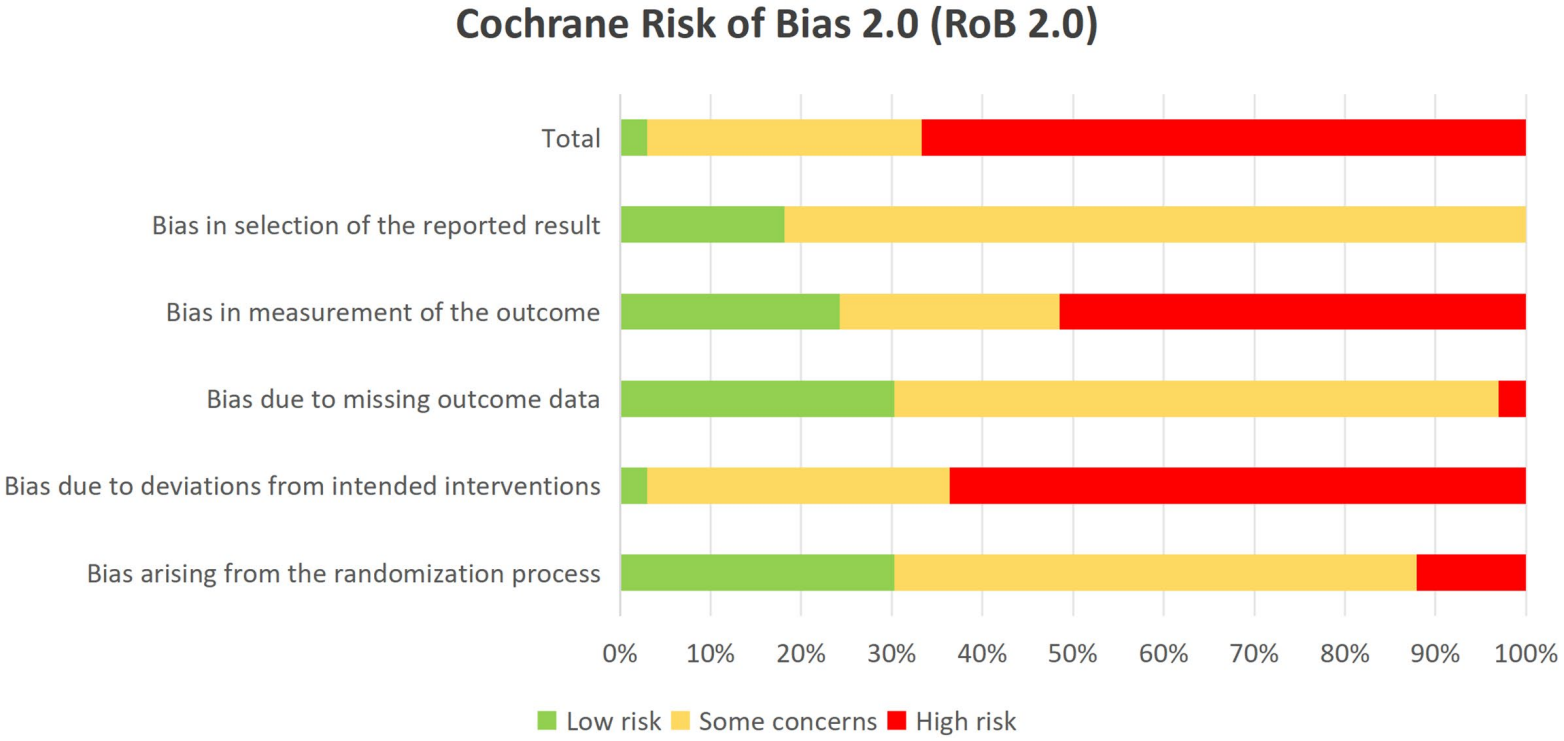
Assessment of bias risk in the included study. Risk of bias assessment “The risk of bias assessment figure shows the evaluation of 33 included studies across five domains: randomization process, intervention delivery, measurement methods, missing data, and selective reporting”.

After evaluation, most studies exhibited varying degrees of bias risk in terms of randomization, intervention implementation, and outcome measurement, while some studies demonstrated higher bias risk due to data missingness or selective reporting. Overall, the majority of included studies showed some degree of bias risk, which may affect the accuracy and consistency of effect estimation. Therefore, although this study provided comparative results of multiple exercise therapies through a network meta-analysis, interpretation of these findings requires caution. Detailed assessment results are presented in Supplementary material, including specific signal question analyses for each study (Figure 2).

### 3.4 Results of network meta-analysis

**Figure 3:**
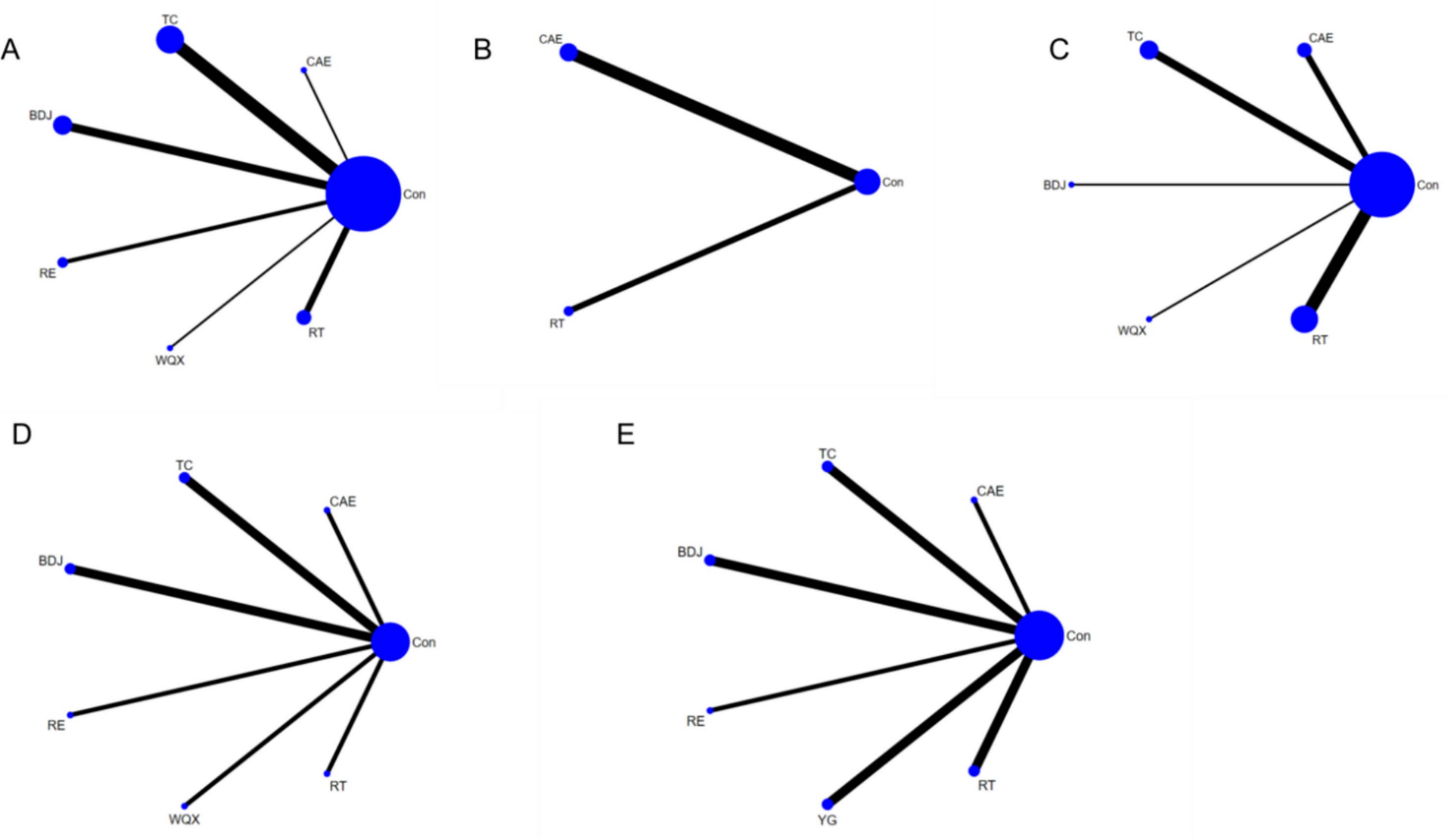
Network diagram of various interventions. **(A)** HAMD; **(B)** SDS; **(C)** BI; **(D)** FMA; **(E)** BBS. Network diagrams of interventions (HAMD, SDS, BI, FMA, BBS). The network diagrams illustrate the comparative relationships among different exercise interventions across various outcomes: HAMD scores for depressive symptoms, SDS scores for self-rated depression, Barthel Index (BI) for daily living abilities, Fugl-Meyer Assessment (FMA) for motor function, and Berg Balance Scale (BBS) for balance ability. Node size represents the total sample size of each intervention group, and edge thickness reflects the number of direct head-to-head comparison studies.

**Figure 4:**
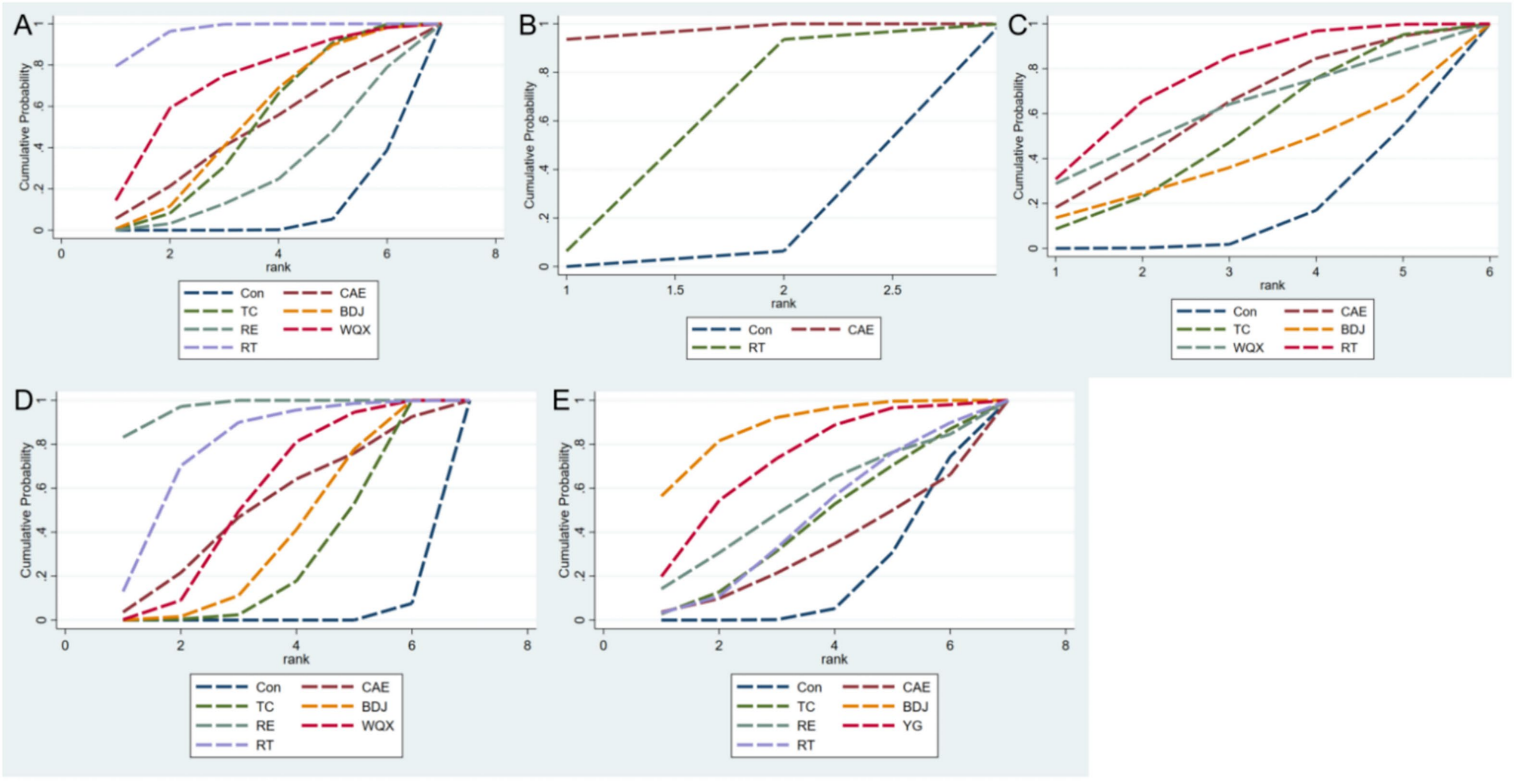
SUCRA score for each intervention measure. **(A)** HAMD; **(B)** SDS; **(C)** BI; **(D)** FMA; **(E)** BBS. SUCRA rankings of interventions (HAMD, SDS, BI, FMA, BBS). The SUCRA rankings display the probability of each exercise intervention being superior for each outcome: HAMD for depressive symptoms, SDS for self-rated depression, BI for daily living abilities, FMA for motor function, and BBS for balance ability. Higher SUCRA values indicate a greater likelihood that the intervention is more effective, providing a clear visual summary of the relative efficacy of all interventions across outcomes.

The 33 included studies covered seven distinct intervention models. Figures 3A–E illustrates the network structure of all competing interventions across various outcome measures. The number of direct head-to-head comparisons between intervention groups is reflected by the thickness of edges, while node diameter is proportional to the total sample size of each intervention group. In addition to main effect estimates, supplementary analyses were conducted to enhance the interpretability and transparency of the network meta-analysis. Meta-regression analysis of age and disease duration across all outcome measures revealed no statistically significant results (*p* > 0.05), indicating that gender and disease duration may not be sources of heterogeneity and demonstrating good transferability. The comparison-adjusted funnel plots did not show significant small-sample effects. Figure 4 and Table 1 present the SUCRA values for all interventions across various outcome measures, providing a clearer and more transparent summary of treatment ranking probabilities.

**Table 1:** The SUCRA values for each treatment modality.

Treatment	CAE	TC	BDJ	RE	WQX	YG	RT
HAMD	47.10%	49.30%	51.70%	28.00%	70.60%	–	95.90%
Rank	5	4	3	6	2	–	1
SDS	96.80%						50.00%
Rank	1	–	–	–	–	–	2
BI	60.60%	50.00%	38.40%		60.70%		75.70%
Rank	3	4	5	–	2	–	1
FMA	50.80%	28.90%	38.60%	96.70%	55.80%	–	77.90%
Rank	4	6	5	1	3	–	2
BBS	31.00%	42.90%	87.80%	53.20%	–	71.90%	44.90%
Rank	6	5	1	3	–	2	4

A higher SUCRA value indicates that the treatment ranks higher in the corresponding outcome metric, while “–” signifies the reporting of relevant data.

#### 3.4.1 Hamilton Depression Rating Scale (HAMD)

Construct network diagrams and perform analysis using a random effects model. HAMD levels were reported in 17 included RCTs (13–29), involving a total of 1,234 participants and evaluating six different exercise therapies (Figure 3A). Meta regression analysis was conducted on gender (*p* = 0.463) and disease duration (*p* = 0.476) in this outcome measure, with results showing no statistical significance (*p* > 0.05). Therefore, these factors were not sources of heterogeneity and demonstrated good transferability. Notably, network meta-analysis revealed that, compared with BDJ [MD = −3.41, 95%CI (−6.24, −0.58)] and TC [MD = −3.02, 95%CI (−5.32, −0.71)], RT significantly reduced HAMD scores. However, no statistically significant difference was observed between rehabilitation training and WQX (*p* > 0.05), as detailed in Supplementary Table S4. SUCRA analysis revealed that RT ranked highest (SUCRA = 95.9%) (Figure 4A), indicating its strongest efficacy among all exercise therapies and significant advantage in reducing HAMD scores. Additionally, although BDJ and TC showed slightly weaker effects, they demonstrated stable moderate effects in sustaining depressive symptom improvement, making them suitable for long-term management or chronic symptom management.

#### 3.4.2 Self-Rating Depression Scale (SDS)

Construct network diagrams and perform analysis using a random effects model. Among the 6 RCTs with included SDS levels reported (23, 30–35), a total of 540 participants were enrolled, evaluating two different exercise therapies (Figure 3B). Meta regression analysis was conducted on gender (*p* = 0.980) in this outcome measure, with results showing no statistical significance (*p* > 0.05). Therefore, this factor was not source of heterogeneity and demonstrated good transferability. But the dataset for this group contains insufficient disease course data to enable analysis. Notably, network meta-analysis revealed that CAE [MD = −12.48, 95%CI (−17.79, −7.17)] significantly reduced SDS levels compared to the control group, whereas no statistically significant difference was observed between CAE and another exercise intervention (*p* > 0.05), details are shown in Supplementary Table S5. SUCRA analysis indicated that aerobic training (SUCRA = 96.8%) was identified as the most effective intervention for improving SDS (Figure 4B). Its advantage lies in suitability for mild-to-moderate depression patients with good cardiopulmonary tolerance who prioritize self-emotional and energy improvement.

#### 3.4.3 Barthel Index (BI)

A network diagram was constructed and analyzed using a random effects model. Among the 15 RCTs with BI level data included (16, 19, 23, 25–27, 29, 31–33, 35–39), a total of 1,146 participants were enrolled, and five different exercise therapies were evaluated (Figure 3C). Meta regression analysis was conducted on gender (*p* = 0.230) and disease duration (*p* = 0.155) in this outcome measure, with results showing no statistical significance (*p* > 0.05). Therefore, these factors were not sources of heterogeneity and demonstrated good transferability. Notably, the network meta-analysis demonstrated that RT significantly improved BI levels compared to the control group [MD = 9.47, 95% CI (2.39, 16.55)], whereas comparisons of other exercise therapies showed no statistically significant differences (*p* > 0.05), as detailed in Supplementary Table S6. The SUCRA analysis revealed that RT (SUCRA = 75.7%) was rated as the most effective intervention for BI improvement (Figure 4C). RT promotes functional recovery through task-oriented training, particularly suitable for patients requiring restoration of daily living abilities. Its advantages in enhancing self-efficacy and life independence were particularly prominent, which contributes to increased patient self-motivation and engagement, further facilitating emotional and functional improvements.

#### 3.4.4 Fugl-Meyer motor function assessment (FMA)

Construct network diagrams and perform analysis using a random effects model. Among the 8 RCTs with FMA level reported (16, 17, 20, 21, 25, 27, 28, 40), a total of 487 participants were enrolled, and six different exercise therapies were evaluated (Figure 3D). Meta regression analysis was conducted on gender (*p* = 0.162) and disease duration (*p* = 0.743) in this outcome measure, with results showing no statistical significance (*p* > 0.05). Therefore, these factors were not sources of heterogeneity and demonstrated good transferability. Compared with WQX [MD = 9.39, 95%CI (2.86, 15.92)], BDJ [MD = 11.60, 95%CI (5.66, 17.54)], and TC [MD = 12.63, 95%CI (6.69, 18.56)], RE significantly improved FMA levels. However, no statistically significant difference was observed between RE and RT (*p* > 0.05), as detailed in Supplementary Table S7. SUCRA analysis revealed that RE (SUCRA = 96.7%) was rated as the most effective intervention for FMA improvement (Figure 4D). Additionally, WQX, BDJ, and TC demonstrated relatively significant benefits, particularly in patients with chronic functional impairments, effectively enhancing their motor function.

#### 3.4.5 Berg balance scale (BBS)

**Figure 5:**
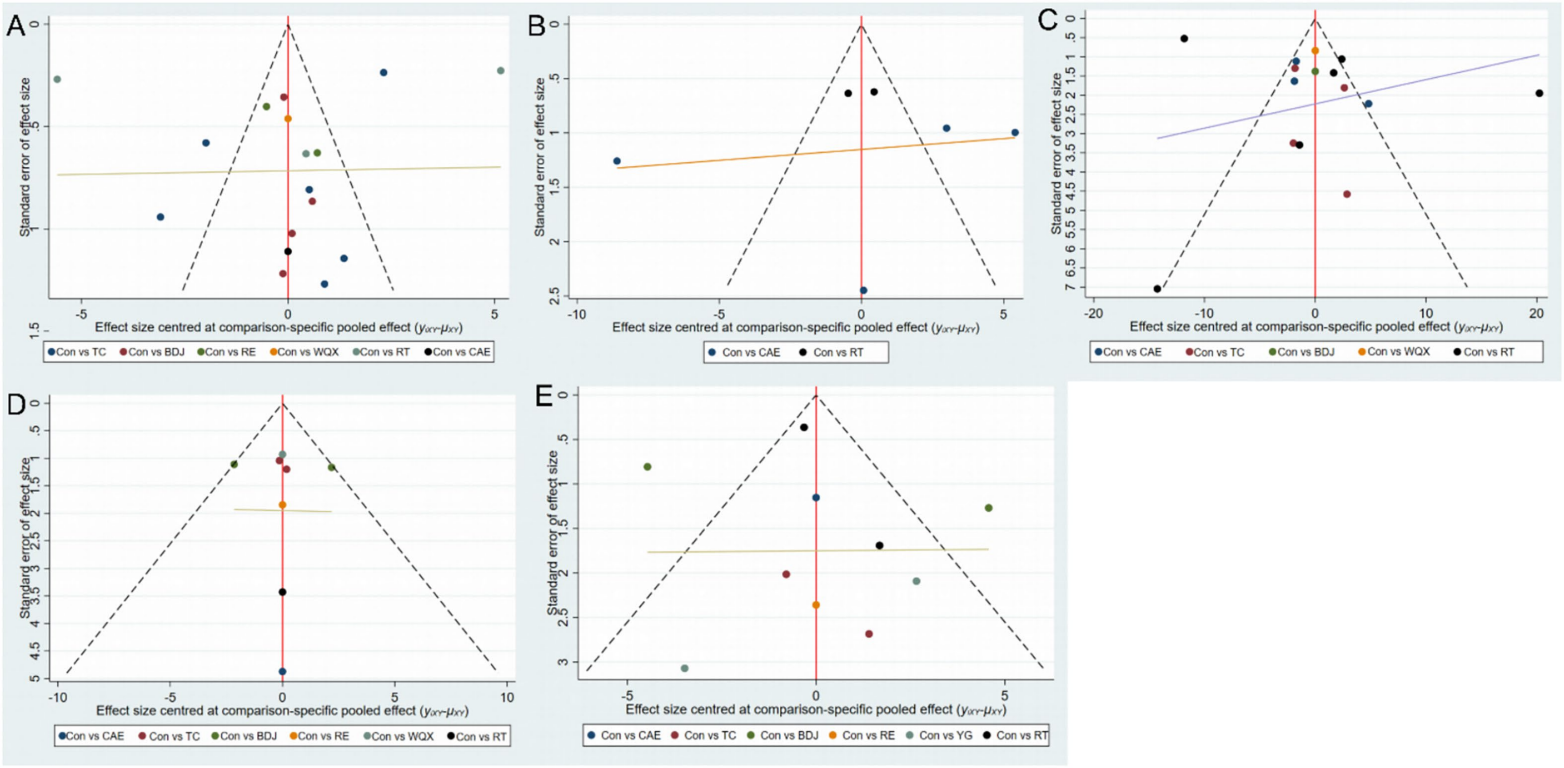
Small study effect funnel plot. **(A)** HAMD; **(B)** SDS; **(C)** BI; **(D)** FMA; **(E)** BBS. The funnel plots assess potential small-study effects or publication bias for the included studies across outcomes: HAMD for depressive symptoms, SDS for self-rated depression, BI for daily living abilities, FMA for motor function, and BBS for balance ability. These plots provide a visual check for asymmetry, helping to identify potential biases that could affect the reliability of the network meta-analysis results.

Construct network diagrams and perform analysis using a random effects model. Among the 10 randomized controlled trials at the BBS level included (20, 22, 29, 37, 39, 41–45), a total of 667 participants were enrolled, and six different exercise therapies were evaluated (Figure 3E). Meta regression analysis was conducted on gender (*p* = 0.856) and disease duration (*p* = 0.185) in this outcome measure, with results showing no statistical significance (*p* > 0.05). Therefore, these factors were not sources of heterogeneity and demonstrated good transferability. Meta-analysis of network data demonstrated that compared with the control group, BDJ [MD = 8.21, 95%CI (2.58, 13.83)] significantly improved BBS levels, whereas comparisons with other exercise therapies showed no statistically significant differences (*p* > 0.05), as detailed in Supplementary Table S8. SUCRA analysis revealed BDJ (SUCRA = 89.3%) as the most effective intervention (Figure 4E), suggesting its potential suitability for long-term balance function management in patients, particularly those with PSD. Although the overall effect was not statistically significant, these exercise therapies enhanced patients’ balance control through balance training, further reinforcing the synergistic relationship between motor function improvement and emotional recovery (Figure 5).

## 4 Discussion

### 4.1 Main findings

This study employed a network meta-analysis framework to systematically compare the effects of various exercise interventions on depressive symptoms and functional outcomes in patients with PSD, and conducted probabilistic ranking of different exercise modalities. The results demonstrated that RT ranked higher in both HAMD score and BI score for daily living activities. CAE showed a certain advantage in SDS score, while RE exhibited significant efficacy in FMA score for activity capacity. Additionally, BDJ demonstrated substantial superiority in BBS score for balance function. These ranking differences among interventions suggest potential differential mechanisms underlying exercise interventions. Unlike previous meta-analyses focusing solely on single modalities, this network meta-analysis integrated multiple exercise modalities, providing comprehensive comparative evidence for efficacy ranking across these modalities. For instance, the latest systematic review indicates that exercise interventions exhibit a moderate improvement effect on depressive symptoms in PSD, while exploring the differential mechanisms underlying the impacts of various exercise modalities (46).

### 4.2 Comparison with previous studies

It should be noted that this study is not the first evidence synthesis study in this field on exercise intervention for improving PSD. Multiple systematic reviews and conventional meta-analyses have previously evaluated the overall efficacy of exercise interventions, preliminarily establishing an evidence base that supports the general benefit of exercise for PSD (46, 47). The aforementioned studies provide crucial evidence for the application of exercise therapy in the management of PSD. However, most of their research designs are based on traditional pairwise comparisons, with analytical focus primarily on the overall judgment of whether exercise is superior to control interventions. Systematic comparisons regarding relative efficacy differences among various exercise modalities, specific advantages across different clinical outcomes, and intervention sequencing remain insufficient (48).

Compared with previous studies, the value of this research lies not in re-proving the “overall efficacy of exercise,” but in further addressing the clinically relevant question of “which exercise may be more suitable for improving specific outcomes.” First, methodologically, this study employed a network meta-analysis to integrate direct and indirect evidence, simultaneously comparing various exercise modalities—including CAE, TC, BDJ, RE, WQX, YG, and RT—expanding the analytical framework previously limited to single-exercise comparisons with controls. Second, in terms of outcome measures, the study not only focused on depressive symptoms reflected by HAMD and SDS but also incorporated functional outcomes such as BI, FMA, and BBS, enabling a more comprehensive evaluation of the differential effects of different exercise interventions on mood improvement, recovery of daily living abilities, enhancement of motor function, and balance control. Third, in terms of result presentation, the SUCRA ranking demonstrated that rehabilitation training performed better in HAMD and BI outcomes, aerobic training showed superiority in SDS outcomes, resistance training ranked highest in FMA outcomes, and Baduanjin exhibited the most prominent performance in BBS outcomes. These findings suggest that the role of exercise therapy in PSD is not homogeneous but may instead reflect an “intervention type-outcome dimension” matching relationship.

Therefore, this study represents an extension and deepening of existing evidence. Previous literature has sufficiently supported the conclusion that exercise interventions confer overall benefits for PSD. The innovative contribution of this study lies in revealing the relative advantages of different exercise modalities across various outcome dimensions through a network meta-analysis, providing more targeted evidence-based foundations for individualized exercise prescriptions in PSD patients. Of course, these comparative results should be interpreted with caution when considering methodological quality, sample size, and network structure characteristics of included studies. Future high-quality randomized controlled trials are still required to further validate optimal application scenarios and long-term efficacy of different exercise modalities.

### 4.3 Interpretation of clinical outcomes

This study demonstrates that different exercise interventions exhibit inconsistent benefit dimensions in PSD-related outcomes, suggesting that the role of exercise therapy in PSD management is not singular or homogeneous. Compared to most previous studies that primarily focused on “whether exercise can improve depressive symptoms,” this study further reveals that different exercise modalities may demonstrate distinct advantages in various clinical outcomes: RT shows superior performance in HAMD and BI outcomes, CAE demonstrates greater efficacy in SDS outcomes, RE ranks highest in FMA outcomes, while BDJ exhibits the most prominent efficacy in BBS outcomes. The clinical significance of this finding lies in the fact that treatment goals for patients with PSD are not entirely uniform. Some patients primarily focus on improving depressive symptoms, while others require more comprehensive rehabilitation targeting daily living skills, motor function, or balance recovery. Therefore, the selection of exercise interventions should emphasize “outcome-oriented” approaches rather than “uniform recommendations” (46–48).

The superior efficacy of RT in HAMD and BI aligns well with the clinical characteristics of stroke rehabilitation. Patients with PSD often experience concurrent emotional distress and functional impairment, where higher levels of functional dependence correlate with more persistent depressive symptoms. RT typically features strong task-oriented approaches and functional specificity, closely mirroring daily activities. Consequently, its benefits may extend beyond direct emotional improvement to indirectly enhance psychological wellbeing by boosting independence and reducing care dependency. This result is consistent with previous observations indicating a “mutual association between functional recovery and emotional improvement,” and suggests that rehabilitation training may be more clinically practical than exercise modalities solely emphasizing physical fitness or emotional regulation in patients with PSD accompanied by significant impairments in activities of daily living (ADL) (4, 5, 49, 50).

The advantages of CAE in SDS outcomes also hold certain clinical interpretative value. Compared with HAMD, SDS places greater emphasis on patients’ subjective perceptions of their emotional and somatic experiences. Therefore, CAE demonstrates superior performance in this outcome, which may suggest its more direct impact on improving subjective energy levels, emotional experiences, and overall state perception. This finding is consistent with previous literature conclusions that “CAE has an overall improving effect on depressive symptoms” (46, 48), and also aligns with the direction of results demonstrating that CAE improves emotional states in patients with PSD in the included studies (31–33). Therefore, CAE may be more suitable for patients with PSD whose chief complaints are low mood, decreased energy, and reduced initiative, and who have acceptable cardiopulmonary tolerance (31–33, 46, 48).

RE ranked highest in FMA outcomes but did not demonstrate equivalent advantages in depressive outcomes, suggesting its primary value may lie in motor function recovery rather than direct intervention on core emotional symptoms. This finding aligns with the inherent training objectives of RE. For stroke patients with muscle weakness, impaired motor control, and limb dysfunction, resistance training is more likely to initially improve strength output and motor performance, thereby yielding more pronounced benefits in FMA outcomes that prioritize motor function. Meanwhile, the limited efficacy of RE in depressive outcomes further indicates that motor function improvement does not necessarily translate into concurrent emotional enhancement, at least during short-term follow-ups. This contrasts with some previous studies that broadly conflated “motor function recovery” with “improved depressive symptoms.” The results of this study suggest that in patients with PSD, RE is more appropriately considered as an intervention primarily aimed at functional recovery, while emotional benefits may be more secondary rather than its primary advantage (24, 28, 49, 51, 52).

Traditional Chinese medicine exercises demonstrated significant “internal heterogeneity” in this study, which represents one of the more valuable findings compared to previous literature. Previous studies often categorized traditional practices such as TC and BDJ as a single group, suggesting their potential benefits for PSD (47). However, this study further revealed inconsistent rankings of different exercises across various outcomes. WQX ranked highest in both HAMD and FMA assessments, while BDJ showed the most prominent performance in BBS. Although TC did not demonstrate optimal results in any single outcome, it exhibited stability across multiple dimensions. These findings indicate that traditional Chinese medicine exercises should not be simplistically regarded as homogeneous interventions but rather differentiated based on their movement structures, training focuses, and clinical objectives. The underlying reasons for these differences may relate to variations in movement amplitude, center of gravity control, coordination training requirements, and participation methods among different exercises, as well as differences in intervention frequency, duration, and baseline functional status among enrolled patients. Therefore, in clinical practice, traditional Chinese medicine exercises are more appropriately understood as an intervention system that “belongs to the same category but with distinct emphases,” rather than a unified protocol that can be mutually substituted (20–23, 25, 37, 39, 47).

In this study, BDJ demonstrated the most prominent performance in the BBS outcome, while YG also ranked relatively high in the SUCRA ranking for this outcome, suggesting that these two types of interventions may have greater potential in improving balance function. This result is largely consistent with previous studies indicating that BDJ and YG contribute to postural control and balance recovery (20, 22, 41, 43). Furthermore, our network comparison study further demonstrates a more pronounced relative advantage of BDJ in this outcome (47).

Overall, the most significant new finding of this study is not the reconfirmation of “exercise’s general benefits for PSD,” but rather the further demonstration that different exercise modalities exhibit varying advantages across distinct clinical outcomes. This discovery shifts the focus of exercise interventions from “whether to use” to “how to select,” bringing PSD exercise therapy closer to the practical needs of individualized rehabilitation. From a clinical perspective, if patients primarily present with depressive symptoms and impaired daily functioning, RT should be prioritized; if subjective emotional experience improvement is more emphasized, CAE may hold greater value; if functional recovery is the primary goal, RE warrants consideration; and if balance disorders are concurrent, BDJ or YG may serve as more appropriate adjunctive options. The aforementioned conclusions are based on the findings of this study as well as previous systematic reviews and related clinical research, but should be interpreted with caution considering factors such as the limited number of included studies, some sparse networks, and high heterogeneity in intervention protocols (41, 43, 46–48, 50).

### 4.4 Clinical significance

The findings of this study suggest that the clinical value of exercise intervention in PSD management is not only reflected in its overall contribution to improving depressive symptoms, but also lies in the differentiated advantages of various exercise modalities across different outcome dimensions. Therefore, the formulation of exercise prescriptions should not be limited to the level of “whether to implement exercise intervention,” but rather should be further individualized based on patients’ primary symptoms, functional impairment characteristics, and rehabilitation goals. For patients with prominent depressive symptoms accompanied by limitations in daily living activities, RT may be a prioritized option with greater comprehensive benefits. For patients exhibiting subjective low mood, decreased energy levels, and reduced activity motivation but with good cardiopulmonary tolerance, CAE may be more suitable as the primary intervention. For patients with significant motor dysfunction whose rehabilitation goals focus on limb function recovery, RE may demonstrate higher clinical value. For patients with concurrent balance impairment or postural control disorders, BDJ and YG can serve as supplementary options worth consideration (41, 43, 46–48, 50).

Meanwhile, this study also suggests that traditional Chinese medicine exercises should not be broadly categorized as a single type of intervention. Although TC, BDJ, and WQX all belong to traditional exercise regimens, their performance in different outcomes varies. Clinical applications should select based on their respective advantages rather than simply substituting one for another. Furthermore, the “intervention type-outcome dimension” matching relationship presented in this study also suggests that motor therapy for PSD should place greater emphasis on multidisciplinary rehabilitation thinking. This means focusing not only on emotional improvement but also on core post-stroke outcomes such as activities of daily living, motor function, and balance ability to enhance overall rehabilitation efficacy (20–23, 37, 39, 47).

It should be emphasized that although this study provides more targeted evidence-based support for individualized exercise interventions in patients with PSD, the results should still be interpreted with caution based on evidence quality. The number of studies included for some outcomes was limited, and some comparisons primarily relied on indirect evidence. Additionally, there were variations in intervention frequency, intensity, duration, and co-interventions across different studies. Therefore, in clinical practice, the development of exercise regimens should still fully consider patient safety, adherence, stroke staging, comorbidities, and accessibility of rehabilitation resources, and be implemented progressively under the guidance of professionals to achieve a balance between efficacy and feasibility (46–48, 50).

### 4.5 Limitations and future directions

Although this study compared the relative efficacy of different exercise therapies for PSD through a network meta-analysis, several limitations remain. First, the methodological quality of included studies was generally limited, with insufficient reporting of randomization, concealed assignment, and blinded implementation in some studies, potentially increasing the risk of bias. Second, the small number of studies included for certain outcomes resulted in a sparse evidence network, leading to reliance on indirect evidence for some comparisons, thereby reducing the precision of results and the stability of rankings. Finally, most studies had short follow-up periods, and evidence regarding long-term efficacy and safety remains inadequate. Future research should conduct multicenter, large-sample, high-quality randomized controlled trials to further standardize exercise prescription design and outcome reporting, while strengthening long-term follow-up to provide more reliable evidence for individualized exercise interventions in post-stroke depression patients.

## 5 Conclusion

The network meta-analysis results of this study indicate that exercise therapy can serve as an effective adjunctive intervention in the comprehensive management of PSD, demonstrating potential benefits in improving depressive symptoms and certain functional outcomes. There were differences in efficacy across various exercise modalities across different outcome dimensions: rehabilitation training showed superior performance in improving HAMD scores and daily living ability (BI), aerobic training exhibited more pronounced advantages in enhancing SDS scores, resistance training ranked highest in promoting motor function recovery (FMA), while Baduanjin demonstrated optimal efficacy in improving balance function (BBS).

The aforementioned results suggest that exercise intervention strategies for patients with PSD should be fully integrated with their primary symptoms, functional impairment characteristics, and rehabilitation goals, adopting outcome-oriented individualized intervention plans rather than uniformly applying a single exercise regimen.

However, given the overall limited methodological quality of the included studies, the small number of studies involving certain outcomes, the relatively sparse evidence network, and the short follow-up periods in most studies, current evidence regarding the relative efficacy and long-term effects of different exercise interventions remains limited. Therefore, future research should focus on conducting more rigorous, multicenter, large-sample, long-term randomized controlled trials to further validate the findings of this study and identify optimal exercise intervention strategies for patients with post-traumatic stress disorder (PSD).

## Data Availability

The original contributions presented in the study are included in the article/Supplementary material, further inquiries can be directed to the corresponding author.

## References

[1] GBD 2021 Stroke Risk Factor Collaborators. Global, regional, and national burden of stroke and its risk factors, 1990-2021: a systematic analysis for the global burden of disease study 2021. Lancet Neurol. (2024) 23:973–1003. doi: 10.1016/S1474-4422(24)00369-7, 39304265 PMC12254192

[2] GBD 2019 Stroke Collaborators. Global, regional, and national burden of stroke and its risk factors, 1990-2019: a systematic analysis for the global burden of disease study 2019. Lancet Neurol. (2021) 20:795–820. doi: 10.1016/S1474-4422(21)00252-0, 34487721 PMC8443449

[3] Liu L Xu M Marshall IJ Wolfe CD Wang Y O'Connell MD. Prevalence and natural history of depression after stroke: a systematic review and meta-analysis of observational studies. PLoS Med. (2023) 20:e1004200. doi: 10.1371/journal.pmed.1004200, 36976794 PMC10047522

[4] Towfighi A Ovbiagele B El Husseini N Hackett ML Jorge RE Kissela BM . Poststroke depression: a scientific statement for healthcare professionals from the American Heart Association/American Stroke Association. Stroke. (2017) 48:e30–43. doi: 10.1161/STR.0000000000000113, 27932603

[5] Robinson RG Jorge RE. Post-stroke depression: a review. Am J Psychiatry. (2016) 173:221–31. doi: 10.1176/appi.ajp.2015.15030363 26684921

[6] Villa RF Ferrari F Moretti A. Post-stroke depression: mechanisms and pharmacological treatment. Pharmacol Ther. (2018) 184:131–44. doi: 10.1016/j.pharmthera.2017.11.005 29128343

[7] Szuhany KL Bugatti M Otto MW. A meta-analytic review of the effects of exercise on brain-derived neurotrophic factor. J Psychiatr Res. (2015) 60:56–64. doi: 10.1016/j.jpsychires.2014.10.003, 25455510 PMC4314337

[8] Kandola A Ashdown-Franks G Hendrikse J Sabiston CM Stubbs B. Physical activity and depression: towards understanding the antidepressant mechanisms of physical activity. Neurosci Biobehav Rev. (2019) 107:525–39. doi: 10.1016/j.neubiorev.2019.09.040, 31586447

[9] Page MJ McKenzie JE Bossuyt PM Boutron I Hoffmann TC Mulrow CD . The PRISMA 2020 statement: an updated guideline for reporting systematic reviews. Syst Rev. (2021) 10:89. doi: 10.1136/bmj.n71 33781348 PMC8008539

[10] Hutton B Salanti G Caldwell DM Chaimani A Schmid CH Cameron C . The PRISMA extension statement for reporting of systematic reviews incorporating network meta-analyses of health care interventions: checklist and explanations. Ann Intern Med. (2015) 162:777–84. doi: 10.7326/M14-2385, 26030634

[11] Sterne JAC Savović J Page MJ Elbers RG Blencowe NS Boutron I . RoB 2: a revised tool for assessing risk of bias in randomised trials. BMJ. (2019) 366:l4898. doi: 10.1136/bmj.l4898 31462531

[12] Chinese Medical Association Neurology Branch, Chinese Medical Association Neurology Branch Cerebrovascular Disease Group. China guidelines for the diagnosis and treatment of acute ischemic stroke 2018. Chin J Neurol. (2018) 51:666–82. doi: 10.3760/cma.j.issn.1006-7876.2018.09.004

[13] Yi S Cuihuan P Jun L Luo Aihua P Shuxiang WX. Effect of weight-loss walking training on efficacy and quality of life in patients with post-ischemic stroke depression. Chinese J Phys Med Rehabil. (2006) 28:387–90. doi: 10.3760/j:issn:0254-1424.2006.06.008

[14] Li Yuling H Xingjuan CL. Clinical observation of seated tai chi exercise in 30 patients with post-stroke depression. Nurs Res. (2012) 26:2254–6. doi: 10.3969/j.issn.1009-6493.2012.24.022

[15] Lin W Qing'an Z Jianyin X Lijie Y Bo Y Hongxia D. Observation on the rehabilitation effect of Tai Chi Chuan on depressive state after cerebral infarction. China Commun Physician (Med Special). (2012) 14:222. doi: 10.3969/j.issn.1007-614x.2012.18.199

[16] Bin Z Qiang T Yan W Luwen Z Huixin Y Tao Y. The effect of tai chi on motor function and depressive state in patients with post-stroke depression. China Rehabil Theory Pract. (2017) 23:334–7. doi: 10.3969/j.issn.1006-9771.2017.03.019

[17] Xiaohui L Ping L Bin X Peng L Yan Z. Clinical study on modified 24-form Tai Chi Chuan for post-stroke depression. Chinese J Health Care Med. (2018) 20:434–5. doi: 10.3969/.issn.1674-3245.2018.05.025

[18] Sun P Zhang S Jiang L Ma Z Yao C Zhu Q . Yijinjing qigong intervention shows strong evidence on clinical effectiveness and electroencephalography signal features for early poststroke depression: a randomized, controlled trial. Front Aging Neurosci. (2022) 14:956316. doi: 10.3389/fnagi.2022.956316, 36034130 PMC9400391

[19] Jian W Wen W Shan Z Hongwen L. Application of Chen-style tai chi training camp in patients with post-stroke depression. China Contemp Med. (2024) 31:58–61,6. doi: 10.3969/j.issn.1674-4721.2024.04.015

[20] Xiaoyu L Wenjie Z Jing L Xiao A Guangrong D. The effect of traditional exercise regimen Baduanjin on post-stroke depression. Chinese J Clin Res Trad Med. (2021) 13:86–8. doi: 10.3969/j.issn.1674-7860.2021.26.027

[21] Zhisheng T Qin L Siyuan L Yalan J Linmei H. Analysis of the effects of Baduanjin exercise on muscle tone and mood in post-stroke depressive patients. China Modern Drug Appl. (2023) 17:169–73. doi: 10.14164/j.cnki.cn11-5581/r.2023.14.048

[22] Zhisheng T Darong Z Siyuan L Ye Y Qin L. Value analysis of Baduanjin combined with balance training intervention for post-stroke depression. Chinese Foreign Med. (2024) 43:15–9. doi: 10.16662/j.cnki.1674-0742.2024.05.015

[23] Yihan L Chen C Hanbin D Mengzhou X Ning Z. Impact of Baduanjin exercise combined with rational emotive behavior therapy on sleep and mood in patients with poststroke depression: a randomized controlled trial. Medicine (Baltimore). (2024) 103:e38180. doi: 10.1097/MD.0000000000038180 38728460 PMC11081619

[24] Xiao L Quanyong G Qiran Z Bao Li H Changhu HL. Effects of progressive resistance training on depression severity and sleep quality in stroke patients with depression. J Xiangyang Vocat Techn College. (2023) 22:103–7. doi: 10.3969/j.issn.2095-6584.2023.01.024

[25] Pengzhen W Jun L Wenzhou L. Observation on the efficacy of Huatuo five-animal exercises training camp in the intervention of post-stroke depression patients. Clin Med Pract. (2024) 33:661–4. doi: 10.16047/j.cnki.cn14-1300/r.2024.09.004

[26] Liping C Ping Y. Effect of exercise therapy on post-stroke depression. J Integr Trad Chinese Western Med Cardiovasc Cerebrovasc Dis. (2006) 3:270–1. doi: 10.3969/j.issn.1672-1349.2006.03.043

[27] Lizhi Y Yi G Qingming Z. The effect of group handicraft activities on upper limb function and emotional state in post-stroke depression patients. China Rehabil. (2022) 37:298–300.

[28] Feng T Zhao C Dong J Xue Z Cai F Li X . The effect of unaffected side resistance training on upper limb function reconstruction and prevention of sarcopenia in stroke patients: a randomized controlled trial. Sci Rep. (2024) 14:25330. doi: 10.1038/S41598-024-76810-2, 39455849 PMC11512051

[29] Stuart M Benvenuti F Macko R Taviani A Segenni L Mayer F . Community-based adaptive physical activity program for chronic stroke: feasibility, safety, and efficacy of the Empoli model. Neurorehabil Neural Repair. (2009) 23:726–34. doi: 10.1177/1545968309332734, 19318465 PMC3024240

[30] Chen Changxiang X Jinxiang ZW Jianmin L Shuxing L Zhiquan C. Effect of Kinect sensory games on depressive mood in stroke patients. Chinese J Behav Med Brain Sci. (2013) 22:619–20. doi: 10.3760/cma.j.issn.1674-6554.2013.07.013

[31] Hua X Wenting J Chengyuan Z Lixia Z Jing G Juan Z . Effects of aerobic training on post-stroke depression patients. Int J Psychiatry. (2023) 50:1072–4. doi: 10.13479/j.cnki.jip.2023.05.028

[32] Jing G Xiaofang X Zheng Juan X Hua ZL Xin Z. Effects of aerobic training on mood, clinical efficacy, and daily living abilities in post-stroke depression patients. Mingyi. (2024) 7:75–7. doi: 10.20255/j.cnki.issn1674-9561.2024.07.029

[33] Jing G Lixia Z Zheng Juan X Hua CS. Effects of moderate-intensity aerobic exercise on elderly patients with mild-to-moderate post-stroke depression. Pract Geriatr. (2025) 39:286–9. doi: 10.3969/j.issn.1003-9198.2025.03.016

[34] Jinlan W Ying C Zhenmei W Chaoping Z Dongmei C Yanjiao Q . Impact of ultra-early activity on rehabilitation and post-stroke depression in patients with acute stroke. Nurs Pract Res. (2018) 15:36–38. doi: 10.3969/j.issn.1672-9676.2018.19.016

[35] Xiuping P Feng T Qianhang Y. Evaluation of the impact of ultra-early activity on rehabilitation and post-stroke depression in patients with acute stroke. World Latest Med Inf Abstr. (2019) 19:89–95. doi: 10.19613/j.cnki.1671-3141.2019.27.050

[36] Jun EM Roh YH Kim MJ. The effect of music-movement therapy on physical and psychological states of stroke patients. J Clin Nurs. (2013) 22:22–31. doi: 10.1111/j.1365-2702.2012.04243.x 22978325

[37] Song R Park M Jang T Oh J Sohn MK. Effects of a tai chi-based stroke rehabilitation program on symptom clusters, physical and cognitive functions, and quality of life: a randomized feasibility study. Int J Environ Res Public Health. (2021) 18:5453. doi: 10.3390/ijerph18105453 34065178 PMC8160714

[38] Vasu DT Mohd Nordin NA Ghazali SE. Effectiveness of autogenic relaxation training in addition to usual physiotherapy on emotional state and functional independence of stroke survivors. Medicine (Baltimore). (2021) 100:e26924., 34414949 10.1097/MD.0000000000026924 PMC8376336

[39] Zhao J Chau JPC Chan AWK Meng Q Choi KC Xiang X . Tailored sitting tai chi program for subacute stroke survivors: a randomized controlled trial. Stroke. (2022) 53:2192–203. doi: 10.1161/STROKEAHA.121.036578, 35387494

[40] Straeten FA van Zyl S Maus B Bauer J Raum H Gross CC . EXERTION: a pilot trial on the effect of aerobic, smartwatch-controlled exercise on stroke recovery: effects on motor function, structural repair, cognition, mental well-being, and the immune system. Neurol Res Pract. (2023) 5:18. doi: 10.1186/S42466-023-00244-W 37170385 PMC10173484

[41] Lai Y-T Lin C-H Hsieh CC Yang J-C Tsou H-H Lin C-C . Combining yoga exercise with rehabilitation improves balance and depression in patients with chronic stroke: a controlled trial. Appl Sci. (2022) 12:922. doi: 10.3390/app12020922

[42] Lai SM Studenski S Richards L Perera S Reker D Rigler S . Therapeutic exercise and depressive symptoms after stroke. J Am Geriatr Soc. (2006) 54:240–7. doi: 10.1111/J.1532-5415.2006.00573.x 16460374

[43] Immink MA Hillier S Petkov J. Randomized controlled trial of yoga for chronic poststroke hemiparesis: motor function, mental health, and quality of life outcomes. Top Stroke Rehabil. (2014) 21:256–71. doi: 10.1310/TSR2103-256, 24985393

[44] Vahlberg B Cederholm T Lindmark B Zetterberg L Hellström K. Short-term and long-term effects of a progressive resistance and balance exercise program in individuals with chronic stroke: a randomized controlled trial. Disabil Rehabil. (2017) 39:1615–22. doi: 10.1080/09638288.2016.1206631, 27415645

[45] Gjellesvik TI Becker F Tjønna AE Indredavik B Lundgaard E Solbakken H . Effects of high-intensity interval training after stroke (the HIIT stroke study) on physical and cognitive function: a multicenter randomized controlled trial. Arch Phys Med Rehabil. (2021) 102:1683–91. doi: 10.1016/J.APMR.2021.05.008, 34102144

[46] Zhang Y Li G Zheng W Xu Z Lv Y Liu X . Effects of exercise on post-stroke depression: a systematic review and Meta-analysis of randomized controlled trials. Life (Basel). (2025) 15:285. doi: 10.3390/life15020285 40003693 PMC11857396

[47] Li Y Li X Huang J Cai H. Effects of traditional Chinese exercises on post-stroke depression: a meta-analysis of randomized controlled trials. Front Public Health. (2025) 13:1570878. doi: 10.3389/fpubh.2025.1570878, 40453492 PMC12125479

[48] Wang ZY Deng YL Zhou TY Jiang ZY Liu Y Liu BF . The effects of exercise interventions on depressive symptoms in stroke patients: a systematic review and meta-analysis. Front Physiol. (2025) 16:1492221. doi: 10.3389/fphys.2025.1492221, 40166715 PMC11955706

[49] Schuch FB Vancampfort D Richards J Rosenbaum S Ward PB Stubbs B. Exercise as a treatment for depression: a meta-analysis adjusting for publication bias. J Psychiatr Res. (2016) 77:42–51. doi: 10.1016/j.jpsychires.2016.02.023, 26978184

[50] Chen R Guo Y Kuang Y Zhang Q. Effects of home-based exercise interventions on post-stroke depression: a systematic review and network meta-analysis. Int J Nurs Stud. (2024) 152:104698. doi: 10.1016/j.ijnurstu.2024.104698, 38290424

[51] Pedersen BK Febbraio MA. Muscles, exercise and obesity: skeletal muscle as a secretory organ. Nat Rev Endocrinol. (2012) 8:457–65. doi: 10.1038/nrendo.2012.49, 22473333

[52] Cotman CW Berchtold NC Christie LA. Exercise builds brain health: key roles of growth factor cascades and inflammation. Trends Neurosci. (2007) 30:464–72. doi: 10.1016/j.tins.2007.06.011, 17765329

