# Supplementary material for: Effectiveness of different types of exercise therapy in improving post-stroke depression: a systematic review and network meta-analysis
Source: Front Med (Lausanne). 2026 May 1;13:1828873. doi: 10.3389/fmed.2026.1828873 (PMC13175843; doi:10.3389/fmed.2026.1828873)

# Appendix

# **Tables**

**Table S1: Search Strategy**

|  | Search | Query |
| --- | --- | --- |
| PubMed | #1 | (post stroke depression OR post stroke depressive disorder OR depression after stroke OR depressive disorder after stroke) |
|  | #2 | ((Exercise Therapy OR Rehabilitation Exercise OR Exercise, Rehabilitation OR Exercises, Rehabilitation OR Rehabilitation Exercises OR Therapy, Exercise OR Exercise Therapies OR Therapies, Exercise OR Remedial Exercise OR Exercise, Remedial OR Exercises, Remedial OR Remedial Exercises) OR (Muscle Stretching Exercises OR Exercise, Muscle Stretching OR Muscle Stretching Exercise OR Active Stretching OR Stretching, Active OR Static-Active Stretching OR Static Active Stretching OR Stretching, Static-Active OR Proprioceptive Neuromuscular Facilitation (PNF) Stretching OR Proprioceptive Neuromuscular Facilitation OR Neuromuscular Facilitation, Proprioceptive OR Proprioceptive Neuromuscular Facilitations OR PNF Stretching OR PNF Stretchings OR Stretching, PNF OR PNF Stretching Exercise OR Exercise, PNF Stretching OR PNF Stretching Exercises OR Stretching Exercise, PNF OR Passive Stretching OR Stretching, Passive OR Static-Passive Stretching OR Static Passive Stretching OR Stretching, Static-Passive OR Relaxed Stretching OR Stretching, Relaxed OR Static Stretching OR Stretching, Static OR Isometric Stretching OR Stretching, Isometric OR Dynamic Stretching OR Stretching, Dynamic OR Ballistic Stretching OR Stretching, Ballistic) OR (Aquatic Therapy OR Hydrotherapy OR Therapy, Aquatic OR Pool Therapy OR Therapy, Pool OR Ai Chi Therapy OR Therapies, Ai Chi OR Therapy, Ai Chi OR Water Tai Chi Therapy OR Aquatic Exercise Therapy OR Exercise Therapy, Aquatic OR Therapy, Aquatic Exercise OR Water Exercise Therapy OR Exercise Therapy, Water OR Therapy, Water Exercise OR Water-based Exercise OR Aqua) OR (Resistance Training OR Training, Resistance OR Strength Training OR Training, Strength OR Weight-Lifting Strengthening Program OR Strengthening Programs, Weight-Lifting OR Strengthening Program, Weight-Lifting OR Weight Lifting Strengthening Program OR Weight-Lifting Strengthening Programs OR Weight-Lifting Exercise Program OR Exercise Programs, Weight-Lifting OR Exercise Program, Weight-Lifting OR Weight Lifting Exercise Program OR Weight-Lifting Exercise Programs OR Weight-Bearing Strengthening Program OR Strengthening Programs, Weight-Bearing OR Strengthening Program, Weight-Bearing OR Weight bearing Strengthening Program OR Weight-Bearing Strengthening Programs OR Weight-Bearing Exercise Program OR Exercise Programs, Weight-Bearing OR Exercise Program, Weight-Bearing OR Weight bearing Exercise Program OR Weight-Bearing Exercise Programs OR Muscle Strength Training) OR ('Yoga' OR 'Tai Ji' OR Tai-ji OR Tai Chi OR Chi, Tai OR Tai Ji Quan OR Ji Quan, Tai OR Quan, Tai Ji OR Taiji OR Taijiquan OR 'T'ai Chi' OR Tai Chi Chuan OR 'Qigong' OR Qi Gong OR Ch'i Kung OR 'Exercise Movement Techniques' OR Movement Techniques, Exercise OR Exercise Movement Technics OR Pilates-Based Exercises OR Exercises, Pilates-Based OR Pilates Based Exercises OR Pilates Training OR Training, Pilates OR McKenzie method OR Heckscher training OR running OR jogging OR exergame OR Swiss balls) OR ('Breathing Exercises' OR Inspiratory muscle training OR Expiratory muscle training OR Exercise, Breathing OR Respiratory Muscle Training OR Muscle Training, Respiratory OR Training, Respiratory Muscle) OR ('Exercise' OR 'High-Intensity Interval Training' OR High Intensity Interval Training OR High-Intensity Interval Trainings OR Interval Training, High-Intensity OR Interval Trainings, High-Intensity OR Training) OR (balance training OR balance trainings OR balance exercise OR balance exercises OR flexibility training OR flexibility trainings OR flexibility exercise OR flexibility exercises OR joint mobility training OR joint mobility trainings OR joint mobility exercise OR joint mobility exercises OR functional movement training OR functional movement trainings OR functional movement exercise OR functional movement exercises OR stability training OR stability trainings OR stability exercise OR stability exercises OR medical gymnastics)) |
|  | #3 | (randomized controlled trial OR controlled clinical trial OR randomized OR placebo OR clinical trials as topic OR RCT) |
|  | #4 | #1 AND #2 AND #3 |
| EMBASE | #1 | ('post stroke depression*':ab,ti,kw OR 'post stroke depressive disorder*':ab,ti,kw OR 'depression after stroke*':ab,ti,kw OR 'depressive disorder after stroke*':ab,ti,kw) |
|  | #2 | ('rehabilitation exercise*':ab,ti,kw OR 'rehabilitation exercises':ab,ti,kw OR 'exercises, rehabilitation':ab,ti,kw OR 'exercises rehabilitation':ab,ti,kw OR 'rehabilitation therapies':ab,ti,kw OR 'therapies, exercise':ab,ti,kw OR 'exercise therapy*':ab,ti,kw OR 'exercise therapies':ab,ti,kw OR 'remedial exercise*':ab,ti,kw OR 'remedial exercises':ab,ti,kw OR 'exercise, remedial':ab,ti,kw OR 'exercises, remedial':ab,ti,kw OR 'muscle stretching exercise*':ab,ti,kw OR 'muscle stretching exercises':ab,ti,kw OR 'exercise, muscle stretching':ab,ti,kw OR 'active stretching':ab,ti,kw OR 'stretching, active':ab,ti,kw OR 'static-active stretching':ab,ti,kw OR 'static active stretching':ab,ti,kw OR 'stretching, static-active':ab,ti,kw OR 'proprioceptive neuromuscular facilitation (PNF) stretching':ab,ti,kw OR 'proprioceptive neuromuscular facilitation':ab,ti,kw OR 'neuromuscular facilitation, proprioceptive':ab,ti,kw OR 'proprioceptive neuromuscular facilitations':ab,ti,kw OR 'PNF stretching':ab,ti,kw OR 'PNF stretchings':ab,ti,kw OR 'stretching, PNF':ab,ti,kw OR 'PNF stretching exercise':ab,ti,kw OR 'exercise, PNF stretching':ab,ti,kw OR 'PNF stretching exercises':ab,ti,kw OR 'stretching exercise, PNF':ab,ti,kw OR 'passive stretching':ab,ti,kw OR 'stretching, passive':ab,ti,kw OR 'static-passive stretching':ab,ti,kw OR 'static passive stretching':ab,ti,kw OR 'stretching, static-passive':ab,ti,kw OR 'relaxed stretching':ab,ti,kw OR 'stretching, relaxed':ab,ti,kw OR 'static stretching':ab,ti,kw OR 'stretching, static':ab,ti,kw OR 'isometric stretching':ab,ti,kw OR 'stretching, isometric':ab,ti,kw OR 'dynamic stretching':ab,ti,kw OR 'stretching, dynamic':ab,ti,kw OR 'ballistic stretching':ab,ti,kw OR 'stretching, ballistic':ab,ti,kw OR 'aquatic therapy':ab,ti,kw OR 'hydrotherapy':ab,ti,kw OR 'therapy, aquatic':ab,ti,kw OR 'pool therapy':ab,ti,kw OR 'therapy, pool':ab,ti,kw OR 'ai chi therapy':ab,ti,kw OR 'therapies, ai chi':ab,ti,kw OR 'therapy, ai chi':ab,ti,kw OR 'water tai chi therapy':ab,ti,kw OR 'aquatic exercise therapy':ab,ti,kw OR 'exercise therapy, aquatic':ab,ti,kw OR 'therapy, aquatic exercise':ab,ti,kw OR 'water exercise therapy':ab,ti,kw OR 'exercise therapy, water':ab,ti,kw OR 'therapy, water exercise':ab,ti,kw OR 'water-based exercise':ab,ti,kw OR 'aqua':ab,ti,kw OR 'resistance training':ab,ti,kw OR 'training, resistance':ab,ti,kw OR 'strength training':ab,ti,kw OR 'training, strength':ab,ti,kw OR 'weight-lifting strengthening program*':ab,ti,kw OR 'strengthening programs, weight-lifting':ab,ti,kw OR 'strengthening program, weight-lifting':ab,ti,kw OR 'weight lifting strengthening program':ab,ti,kw OR 'weight-lifting strengthening programs':ab,ti,kw OR 'weight-lifting exercise program*':ab,ti,kw OR 'exercise programs, weight-lifting':ab,ti,kw OR 'exercise program, weight-lifting':ab,ti,kw OR 'weight lifting exercise program':ab,ti,kw OR 'weight-lifting exercise programs':ab,ti,kw OR 'weight-bearing strengthening program*':ab,ti,kw OR 'strengthening programs, weight-bearing':ab,ti,kw OR 'strengthening program, weight-bearing':ab,ti,kw OR 'weight bearing strengthening program':ab,ti,kw OR 'weight-bearing strengthening programs':ab,ti,kw OR 'weight-bearing exercise program*':ab,ti,kw OR 'exercise programs, weight-bearing':ab,ti,kw OR 'exercise program, weight-bearing':ab,ti,kw OR 'weight bearing exercise program':ab,ti,kw OR 'weight-bearing exercise programs':ab,ti,kw OR 'muscle strength training':ab,ti,kw OR 'yoga':ab,ti,kw OR 'tai ji':ab,ti,kw OR 'tai-ji':ab,ti,kw OR 'tai chi':ab,ti,kw OR 'chi, tai':ab,ti,kw OR 'tai ji quan':ab,ti,kw OR 'ji quan, tai':ab,ti,kw OR 'quan, tai ji':ab,ti,kw OR 'taiji':ab,ti,kw OR 'taijiquan':ab,ti,kw OR 'tai chi':ab,ti,kw OR 'tai chi chuan':ab,ti,kw OR 'qigong':ab,ti,kw OR 'qi gong':ab,ti,kw OR 'chi kung':ab,ti,kw OR 'exercise movement techniques':ab,ti,kw OR 'movement techniques, exercise':ab,ti,kw OR 'exercise movement technics':ab,ti,kw OR 'pilates-based exercises':ab,ti,kw OR 'exercises, pilates-based':ab,ti,kw OR 'pilates based exercises':ab,ti,kw OR 'pilates training':ab,ti,kw OR 'training, pilates':ab,ti,kw OR 'mckenzie method':ab,ti,kw OR 'heckscher training':ab,ti,kw OR 'running':ab,ti,kw OR 'jogging':ab,ti,kw OR 'exergame':ab,ti,kw OR 'swiss balls':ab,ti,kw OR 'breathing exercises':ab,ti,kw OR 'inspiratory muscle training':ab,ti,kw OR 'expiratory muscle training':ab,ti,kw OR 'exercise, breathing':ab,ti,kw OR 'respiratory muscle training':ab,ti,kw OR 'muscle training, respiratory':ab,ti,kw OR 'training, respiratory muscle':ab,ti,kw OR 'exercise':ab,ti,kw OR 'high-intensity interval training':ab,ti,kw OR 'high intensity interval training':ab,ti,kw OR 'high-intensity interval trainings':ab,ti,kw OR 'interval training, high-intensity':ab,ti,kw OR 'interval trainings, high-intensity':ab,ti,kw OR 'training':ab,ti,kw OR 'balance training':ab,ti,kw OR 'balance trainings':ab,ti,kw OR 'balance exercise':ab,ti,kw OR 'balance exercises':ab,ti,kw OR 'flexibility training':ab,ti,kw OR 'flexibility trainings':ab,ti,kw OR 'flexibility exercise':ab,ti,kw OR 'flexibility exercises':ab,ti,kw OR 'joint mobility training':ab,ti,kw OR 'joint mobility trainings':ab,ti,kw OR 'joint mobility exercise':ab,ti,kw OR 'joint mobility exercises':ab,ti,kw OR 'functional movement training':ab,ti,kw OR 'functional movement trainings':ab,ti,kw OR 'functional movement exercise':ab,ti,kw OR 'functional movement exercises':ab,ti,kw OR 'stability training':ab,ti,kw OR 'stability trainings':ab,ti,kw OR 'stability exercise':ab,ti,kw OR 'stability exercises':ab,ti,kw OR 'medical gymnastics':ab,ti,kw) |
|  | #3 | (randomized*:ab,ti,kw OR controlled clinical trial*:ab,ti,kw OR random*:ab,ti,kw OR placebo*:ab,ti,kw OR RCT:ab,ti,kw) |
|  | #4 | #1 AND #2 AND #3 |
| Web of science | #1 | TS=(post stroke depression OR post stroke depressive disorder OR depression after stroke OR depressive disorder after stroke) |
|  | #2 | TS=((Exercise Therapy OR Rehabilitation Exercise OR Exercise, Rehabilitation OR Exercises, Rehabilitation OR Rehabilitation Exercises OR Therapy, Exercise OR Exercise Therapies OR Therapies, Exercise OR Remedial Exercise OR Exercise, Remedial OR Exercises, Remedial OR Remedial Exercises) OR (Muscle Stretching Exercises OR Exercise, Muscle Stretching OR Muscle Stretching Exercise OR Active Stretching OR Stretching, Active OR Static-Active Stretching OR Static Active Stretching OR Stretching, Static-Active OR Proprioceptive Neuromuscular Facilitation (PNF) Stretching OR Proprioceptive Neuromuscular Facilitation OR Neuromuscular Facilitation, Proprioceptive OR Proprioceptive Neuromuscular Facilitations OR PNF Stretching OR PNF Stretchings OR Stretching, PNF OR PNF Stretching Exercise OR Exercise, PNF Stretching OR PNF Stretching Exercises OR Stretching Exercise, PNF OR Passive Stretching OR Stretching, Passive OR Static-Passive Stretching OR Static Passive Stretching OR Stretching, Static-Passive OR Relaxed Stretching OR Stretching, Relaxed OR Static Stretching OR Stretching, Static OR Isometric Stretching OR Stretching, Isometric OR Dynamic Stretching OR Stretching, Dynamic OR Ballistic Stretching OR Stretching, Ballistic) OR (Aquatic Therapy OR Hydrotherapy OR Therapy, Aquatic OR Pool Therapy OR Therapy, Pool OR Ai Chi Therapy OR Therapies, Ai Chi OR Therapy, Ai Chi OR Water Tai Chi Therapy OR Aquatic Exercise Therapy OR Exercise Therapy, Aquatic OR Therapy, Aquatic Exercise OR Water Exercise Therapy OR Exercise Therapy, Water OR Therapy, Water Exercise OR Water-based Exercise OR Aqua) OR (Resistance Training OR Training, Resistance OR Strength Training OR Training, Strength OR Weight-Lifting Strengthening Program OR Strengthening Programs, Weight-Lifting OR Strengthening Program, Weight-Lifting OR Weight Lifting Strengthening Program OR Weight-Lifting Strengthening Programs OR Weight-Lifting Exercise Program OR Exercise Programs, Weight-Lifting OR Exercise Program, Weight-Lifting OR Weight Lifting Exercise Program OR Weight-Lifting Exercise Programs OR Weight-Bearing Strengthening Program OR Strengthening Programs, Weight-Bearing OR Strengthening Program, Weight-Bearing OR Weight bearing Strengthening Program OR Weight-Bearing Strengthening Programs OR Weight-Bearing Exercise Program OR Exercise Programs, Weight-Bearing OR Exercise Program, Weight-Bearing OR Weight bearing Exercise Program OR Weight-Bearing Exercise Programs OR Muscle Strength Training) OR ('Yoga' OR 'Tai Ji' OR Tai-ji OR Tai Chi OR Chi, Tai OR Tai Ji Quan OR Ji Quan, Tai OR Quan, Tai Ji OR Taiji OR Taijiquan OR 'T'ai Chi' OR Tai Chi Chuan OR 'Qigong' OR Qi Gong OR Ch'i Kung OR 'Exercise Movement Techniques' OR Movement Techniques, Exercise OR Exercise Movement Technics OR Pilates-Based Exercises OR Exercises, Pilates-Based OR Pilates Based Exercises OR Pilates Training OR Training, Pilates OR McKenzie method OR Heckscher training OR running OR jogging OR exergame OR Swiss balls) OR ('Breathing Exercises' OR Inspiratory muscle training OR Expiratory muscle training OR Exercise, Breathing OR Respiratory Muscle Training OR Muscle Training, Respiratory OR Training, Respiratory Muscle) OR ('Exercise' OR 'High-Intensity Interval Training' OR High Intensity Interval Training OR High-Intensity Interval Trainings OR Interval Training, High-Intensity OR Interval Trainings, High-Intensity OR Training) OR (balance training OR balance trainings OR balance exercise OR balance exercises OR flexibility training OR flexibility trainings OR flexibility exercise OR flexibility exercises OR joint mobility training OR joint mobility trainings OR joint mobility exercise OR joint mobility exercises OR functional movement training OR functional movement trainings OR functional movement exercise OR functional movement exercises OR stability training OR stability trainings OR stability exercise OR stability exercises OR medical gymnastics)) |
|  | #3 | TS=(randomized controlled trial OR controlled clinical trial OR randomized OR placebo OR clinical trials as topic OR RCT) |
|  | #4 | #1 AND #2 AND #3 |
| Cochrane Library | #1 | ((Exercise Therapy OR Rehabilitation Exercise OR Exercise, Rehabilitation OR Exercises, Rehabilitation OR Rehabilitation Exercises OR Therapy, Exercise OR Exercise Therapies OR Therapies, Exercise OR Remedial Exercise OR Exercise, Remedial OR Exercises, Remedial OR Remedial Exercises) OR (Muscle Stretching Exercises OR Exercise, Muscle Stretching OR Muscle Stretching Exercise OR Active Stretching OR Stretching, Active OR Static - Active Stretching OR Static Active Stretching OR Stretching, Static - Active OR Proprioceptive Neuromuscular Facilitation (PNF) Stretching OR Proprioceptive Neuromuscular Facilitation OR Neuromuscular Facilitation, Proprioceptive OR Proprioceptive Neuromuscular Facilitations OR PNF Stretching OR PNF Stretchings OR Stretching, PNF OR PNF Stretching Exercise OR Exercise, PNF Stretching OR PNF Stretching Exercises OR Stretching Exercise, PNF OR Passive Stretching OR Stretching, Passive OR Static - Passive Stretching OR Static Passive Stretching OR Stretching, Static - Passive OR Relaxed Stretching OR Stretching, Relaxed OR Static Stretching OR Stretching, Static OR Isometric Stretching OR Stretching, Isometric OR Dynamic Stretching OR Stretching, Dynamic OR Ballistic Stretching OR Stretching, Ballistic) OR (Aquatic Therapy OR Hydrotherapy OR Therapy, Aquatic OR Pool Therapy OR Therapy, Pool OR Ai Chi Therapy OR Therapies, Ai Chi OR Therapy, Ai Chi OR Water Tai Chi Therapy OR Aquatic Exercise Therapy OR Exercise Therapy, Aquatic OR Therapy, Aquatic Exercise OR Water Exercise Therapy OR Exercise Therapy, Water OR Therapy, Water Exercise OR Water - based Exercise OR Aqua) OR (Resistance Training OR Training, Resistance OR Strength Training OR Training, Strength OR Weight - Lifting Strengthening Program OR Strengthening Programs, Weight - Lifting OR Strengthening Program, Weight - Lifting OR Weight Lifting Strengthening Program OR Weight - Lifting Strengthening Programs OR Weight - Lifting Exercise Program OR Exercise Programs, Weight - Lifting OR Exercise Program, Weight - Lifting OR Weight Lifting Exercise Program OR Weight - Lifting Exercise Programs OR Weight - Bearing Strengthening Program OR Strengthening Programs, Weight - Bearing OR Strengthening Program, Weight - Bearing OR Weight bearing Strengthening Program OR Weight - Bearing Strengthening Programs OR Weight - Bearing Exercise Program OR Exercise Programs, Weight - Bearing OR Exercise Program, Weight - Bearing OR Weight bearing Exercise Program OR Weight - Bearing Exercise Programs OR Muscle Strength Training) OR ('Yoga' OR 'Tai Ji' OR Tai - ji OR Tai Chi OR Chi, Tai OR Tai Ji Quan OR Ji Quan, Tai OR Quan, Tai Ji OR Taiji OR Taijiquan OR 'T'ai Chi' OR Tai Chi Chuan OR 'Qigong' OR Qi Gong OR Ch'i Kung OR 'Exercise Movement Techniques' OR Movement Techniques, Exercise OR Exercise Movement Technics OR Pilates - Based Exercises OR Exercises, Pilates - Based OR Pilates Based Exercises OR Pilates Training OR Training, Pilates OR McKenzie method OR Heckscher training OR running OR jogging OR exergame OR Swiss balls) OR ('Breathing Exercises' OR Inspiratory muscle training OR Expiratory muscle training OR Exercise, Breathing OR Respiratory Muscle Training OR Muscle Training, Respiratory OR Training, Respiratory Muscle) OR ('Exercise' OR 'High - Intensity Interval Training' OR High Intensity Interval Training OR High - Intensity Interval Trainings OR Interval Training, High - Intensity OR Interval Trainings, High - Intensity OR Training) OR (balance training OR balance trainings OR balance exercise OR balance exercises OR flexibility training OR flexibility trainings OR flexibility exercise OR flexibility exercises OR joint mobility training OR joint mobility trainings OR joint mobility exercise OR joint mobility exercises OR functional movement training OR functional movement trainings OR functional movement exercise OR functional movement exercises OR stability training OR stability trainings OR stability exercise OR stability exercises OR medical gymnastics)) |
|  | #2 | (post stroke depression OR post stroke depressive disorder OR depression after stroke OR depressive disorder after stroke) |
|  | #3 | (randomized controlled trial OR controlled clinical trial OR randomized OR placebo OR clinical trials as topic OR RCT) |
|  | #4 | #1 AND #2 AND #3 |
| CNKI | #1 | （运动疗法 or 康复运动 or 运动疗法 or 康复运动疗法 or 矫正运动 or 肌肉拉伸运动 or 运动 or 主动拉伸 or 静态主动拉伸 or 拉伸 or 本体感觉神经肌肉促进拉伸 or 被动拉伸 or 放松拉伸 or 静态拉伸 or 等长拉伸 or 弹震拉伸 or 水疗 or 太极 or 抗阻训练 or 力量训练 or举重强化计划 or 普拉提 or 跑步 or 活力游戏 or 瑞士球 or 呼吸练习 or 高强度间歇训练 or 平衡训练） |
|  | #2 | （卒中后 or 脑梗死后 or 脑血管意外后 or 脑缺血后） |
|  | #3 | （抑郁） |
|  | #4 | #1 AND #2 AND #3 |
| WanFang | #1 | 主题：(运动疗法 or 康复运动 or 运动疗法 or 康复运动疗法 or 矫正运动 or 肌肉拉伸运动 or 运动 or 主动拉伸 or 静态主动拉伸 or 拉伸 or 本体感觉神经肌肉促进拉伸 or 被动拉伸 or 放松拉伸 or 静态拉伸 or 等长拉伸 or 弹震拉伸 or 水疗 or 太极 or 抗阻训练 or 力量训练 or举重强化计划 or 普拉提 or 跑步 or 活力游戏 or 瑞士球 or 呼吸练习 or 高强度间歇训练 or 平衡训练) |
|  | #2 | 主题：(卒中后 or 脑梗死后 or 脑血管意外后 or 脑缺血后) |
|  | #3 | 主题：(抑郁) |
|  | #4 | #1 AND #2 AND #3 |
| VIP | #1 | M=(运动疗法 or 康复运动 or 康复运动疗法 or 矫正运动 or 肌肉拉伸运动 or 运动 or 主动拉伸 or 静态动拉伸 or 拉伸 or 本体感觉神经肌肉促进拉伸 or 被动拉伸 or 放松拉伸 or 静态拉伸 or 等长拉伸 or 弹震拉伸 or 水疗 or 太极 or 抗阻训练 or 力量训练 or 举重强化计划 or 普拉提 or 跑歩 or 活カ游戏 or 呯吸练习 or 髙强度间歇训练 or 平衡训练) |
|  | #2 | M=(卒中后 or 脑梗死后 or 脑血管意外后 or 脑缺血后) |
|  | #3 | M=(抑郁) |
|  | #4 | #1 AND #2 AND #3 |
| CBM | #1 | "运动疗法"[不加权:扩展] |
|  | #2 | "康复运动"[常用字段:智能] OR "康复运动疗法"[常用字段:智能] OR "矫正运动"[常用字段:智能] OR "肌肉拉伸运动"[常用字段:智能] OR "运动"[常用字段:智能] OR "主动拉伸"[常用字段:智能] OR "静态动拉伸"[常用字段:智能] OR "拉伸"[常用字段:智能] OR "本体感觉神经肌肉促进拉伸"[常用字段:智能] |
|  | #3 | "被动拉伸"[常用字段:智能] OR "放松拉伸"[常用字段:智能] OR "静态拉伸"[常用字段:智能] OR "等长拉伸"[常用字段:智能] OR "弹震拉伸"[常用字段:智能] OR "水疗"[常用字段:智能] OR "太极"[常用字段:智能] OR "抗阻训练"[常用字段:智能] OR "力量训练"[常用字段:智能] |
|  | #4 | "举重强化计划"[常用字段:智能] OR "普拉提"[常用字段:智能] OR "跑歩"[常用字段:智能] OR "活力游戏"[常用字段:智能] OR "呯吸练习"[常用字段:智能] OR "髙强度间歇训练"[常用字段:智能] OR "平衡训练"[常用字段:智能] |
|  | #5 | "卒中后"[常用字段:智能] OR "脑梗死后"[常用字段:智能] OR "脑血管意外后"[常用字段:智能] OR "脑缺血后"[常用字段:智能] |
|  | #6 | "抑郁"[不加权:扩展] AND "抑郁症"[不加权:扩展] AND "抑郁"[不加权:扩展] AND "抑郁"[不加权:扩展] |
|  | #7 | #1 or #2 or #3 or #4 |
|  | #8 | #5 and #6 and #7 |

**Table S2: Overview Table of the sample size distribution across different therapeutic categories**

| Overview Table 2 | | |
| --- | --- | --- |
| Intervention characteristics | total number of studies | total sample size |
| CAE | 7 | 472 |
| TC | 8 | 565 |
| BDJ | 4 | 300 |
| RE | 3 | 254 |
| WQX | 1 | 82 |
| YG | 2 | 57 |
| RT | 8 | 609 |
| Total | 33 | 2339 |

Note:CAE, aerobic exercise;TC,Tai Chi;BDJ,Baduanjin;RE,resistance training; WQX, Wuqinxi;YG,yoga;RT,rehabilitation training.

# **Table S3:Baseline table of included studies**

| Author（year） | Sample size(n) | | Sex(male) | | Age(years) | | Disease course（months) | | Intervention time(months) | | Intervention characteristics | | out-comes | Intervention frequency(times/week） | total frequency | duration per session (minutes) |
| --- | --- | --- | --- | --- | --- | --- | --- | --- | --- | --- | --- | --- | --- | --- | --- | --- |
|  | T | C | T | C | T | C | T | C | T | C | T | C |  |  |  |  |
| Chen Changxiang 2013 | 30 | 30 | 24 | 22 | 52.93±6.20 | 51.67±7.18 |  |  | 1 | 1 | CAE | Conventional rehabilitation | SDS | 3 | 12 | 30 |
| Guo Jing 2024 | 50 | 50 | 29 | 24 | 61.20±4.80 | 61.60±5.00 |  |  | 1 | 1 | CAE | Conventional rehabilitation | SDS,BI | 5 | 20 | 30 |
| Guo Jing 2025 | 30 | 30 | 19 | 17 | 66.18±5.12 | 65.41±4.82 | 2.43±1.05 | 1.98±1.33 | 1 | 1 | CAE | Conventional rehabilitation | SDS,BI | 5 | 20 | 30 |
| Shen Yi 2006 | 30 | 30 | 17 | 16 | 61.00±5.60 | 62.00±4.50 | 1.23 | 1.2 | 1 | 1 | CAE | Conventional rehabilitation | HAMD,FMA,BI | 7 | 28 | 30 |
| Xu Hua 2023 | 50 | 50 | 29 | 31 | 63.71±4.81 | 64.25±4.76 | 1.70±0.36 | 1.69±0.35 | 3 | 3 | CAE | Conventional rehabilitation | SDS,BI | 3 | 36 | 30 |
| TorIvar Gjellesvik 2021 | 36 | 34 |  |  | 57.60±9.20 | 58.70±9.20 | 60 | 60 | 2 | 2 | CAE | Conventional rehabilitation | BBS | 3 | 24 | 60 |
| Frederike A. Straeten 2023 | 15 | 15 |  |  |  |  | 0.5 | 0.5 | 3 | 3 | CAE | Conventional rehabilitation | FMA | 5 | 60 | 45 |
| PingPingSun 2022 | 30 | 30 | 17 | 17 | 62.70±8.63 | 65.37±6.52 | 7.97±5.27 | 7.53±4.09 | 0.75 | 0.75 | TC | routine segmental rehabilitation training | HAMD | 7 | 21 | 60 |
| Li Xiaohui 2018 | 30 | 30 | 17 | 17 | 71.03±8.21 | 71.06±8.33 | 3.69±0.46 | 3.42±0.46 | 3 | 3 | TC | Oral aspirin and atorvastatin | HAMD,FMA | 7 | 84 | 60 |
| Li Yuling 2012 | 36 | 32 | 17 | 12 | 38-76 | 38-76 | 0.5 | 0.5 | 1.25 | 1.25 | TC | Conventional rehabilitation | HAMD | 3 | 36 | 30 |
| Wang Jian 2024 | 40 | 40 | 22 | 24 | 56.86±3.37 | 56.35±3.35 | 6.18±0.82 | 6.25±0.85 | 2 | 2 | TC | Conventional rehabilitation | HAMD,BI,FMA | 7 | 56 | 60 |
| Wang Lin 2012 | 36 | 33 | 20 | 22 | 55.80±3.54 | 51.20±7.80 |  |  | 1 | 1 | TC | Conventional rehabilitation | HAMD | 2 | -- | -- |
| Zhao Bin 2017 | 30 | 30 | 20 | 19 | 53.85±11.69 | 51.38±14.83 | 1.35±0.77 | 1.41±0.66 | 2 | 2 | TC | Conventional rehabilitation | HAMD,BI,FMA | 5 | 40 | 30 |
| Jie Zhao 2022 | 69 | 65 | 36 | 31 | 62.61±12.88 | 63.35±12.90 | 1.65±1.60 | 1.53±1.63 | 3 | 3 | TC | Conventional rehabilitation | BI,BBS | 3 | 36 | 40 |
| Rhayun Song 2021 | 18 | 16 | 10 | 11 | 58.72±17.13 | 57.18±10.65 | 7.58±5.98 | 10.94±8.50 | 6 | 6 | TC | Conventional rehabilitation | BI,BBS | 1 | 24 | 30 |
| Yihan 2024 | 50 | 50 | 31 | 29 | 58.86±10.83 | 56.22±11.54 | 2.84±1.47 | 2.60±1.31 | 2 | 2 | BDJ | REBT | HAMD,BI | 14 | 112 | 30 |
| Liu Xiaoyu 2021 | 30 | 30 | 11 | 10 | 57.58±5.71 | 56.85±7.47 | 3.85±1.41 | 3.90±1.53 | 1 | 1 | BDJ | Oral escitalopram | HAMD,FMA | 3 | 12 | 45 |
| Tang Zhisheng 2023 | 30 | 30 | 17 | 18 | 57.18±7.71 | 57.14±7.68 | 4.16±1.65 | 4.13±1.32 | 2 | 2 | BDJ | Conventional medication | HAMD,FMA | 14 | 96 | 15 |
| Tang Zhisheng 2024 | 40 | 40 | 23 | 22 | 54.19±5.62 | 53.68±5.38 | 6.13±2.57 | 5.98±2.35 | 3 | 3 | BDJ | Balance training | HAMD,BBS | 7 | 90 |  |
| Liu Xiao 2023 | 55 | 55 | 33 | 32 | 56.01±2.13 | 54.01±1.08 | 0.34±0.06 | 0.34±0.03 | 2 | 2 | RE | Conventional rehabilitation | HAMD | 3 | 24 | 30 |
| Birgit Vahlberg 2016 | 34 | 33 | 27 | 24 | 72.60±5.50 | 73.70±5.30 | 36 | 36 | 3 | 3 | RE | Conventional rehabilitation | BBS | 2 | 24 | 60 |
| Taisheng Feng 2024 | 38 | 39 | 27 | 31 | 57.03±13.39 | 56.54±10.23 | 2 | 2 | 1 | 1 | RE | Conventional rehabilitation | HAMD,FMA | 5 | 20 | 60 |
| Wen Pengzhen 2024 | 41 | 41 | 24 | 28 | 62.53±3.35 | 62.59±3.37 | 10.64±2.27 | 10.59±2.23 | 2 | 2 | WQX | Conventional rehabilitation | HAMD,BI,FMA | 5 | 40 | 30 |
| Yen 2022 | 16 | 19 | 10 | 10 | 56.80±9.11 | 60.90±10.74 | 18.69±17.95 | 26.75±38.43 | 2 | 2 | YG | Conventional rehabilitation | BBS | 2 | 16 | 60 |
| Maarten A.Immink 2014 | 11 | 11 | 6 | 3 | 56.10±13.60 | 63.20±17.40 | 81.60±77.50 | 23.30±12.50 | 2.5 |  | YG |  | BBS | 1 | 10 | 90 |
| Chen Liping 2006 | 40 | 40 | 22 | 24 | 56.20±9.20 | 59.30±9.60 |  |  | 2 | 2 | RT | Conventional drug therapy | HAMD,BI,FMA | 7 | 56 | 40 |
| Pang Xiuping 2019 | 50 | 50 |  |  |  |  |  |  | 0.8 | 0.8 | RT | Conventional rehabilitation | SDS,BI | 14 |  | 30 |
| Wang Jinlan 2018 | 60 | 60 | 40 | 39 | 62.41±1.31 | 63.97±1.37 |  |  | 3 | 3 | RT | Conventional rehabilitation | SDS,BI | 14 |  | 30 |
| Yang Lizhi 2022 | 29 | 29 | 15 | 14 | 54.41±7.61 | 53.32±7.82 | 0.97±0.12 | 0.96±0.12 | 1 | 1 | RT | Conventional rehabilitation | HAMD,BI,FMA | 5 | 20 | 30 |
| Sue-Min Lai 2006 | 44 | 49 | 23 | 27 | 68.50±9.00 | 70.40±11.30 | 2.58±0.96 | 2.47±0.91 | 3 | 3 | RT | Conventional rehabilitation | BBS | 3 | 36 |  |
| Mary Stuart 2009 | 40 | 38 | 25 | 29 | 66.80±1.40 | 70.00±1.70 | 50.40±9.60 | 42.00±6.00 | 6 |  | RT | Conventional nursing | HAMD，BBS | 3 | 72 | 60 |
| Eun-Mi Jun 2012 | 15 | 15 | 9 | 9 | 60.70±8.59 | 55.10±17.23 | 0.5 | 0.5 | 2 |  | RT | Conventional nursing | BI | 3 | 24 | 60 |
| Deepak Thazhakkattu Vasu 2021 | 25 | 25 |  |  | 40-75 | 40-75 | 3 | 3 | 3 | 3 | RT | Conventional rehabilitation | BI | 3 | 36 | 60 |

Note:CAE, aerobic exercise;TC,Tai Chi;BDJ,Baduanjin;RE,resistance training; WQX, Wuqinxi;YG,yoga;RT,rehabilitation training;HAMD,Hamilton Depression Rating Scale;SDS,Self-Rating Depression Scale;BI,the Barthel Index;FMA,the Fugl-Meyer Assessment;BBS,the Berg Balance Scale.

**Table S4: Effects of Different Exercise Therapies on HAMD**

| RT |  |  |  |  |  |  |
| --- | --- | --- | --- | --- | --- | --- |
| -2.90 (-9.19,3.40) | WQX |  |  |  |  |  |
| -5.04 (-9.27,-0.81) | -2.14 (-8.28,4.01) | BDJ |  |  |  |  |
| -5.43 (-9.33,-1.54) | -2.53 (-8.46,3.39) | -0.40 (-4.05,3.26) | TC |  |  |  |
| -5.45 (-12.05,1.15) | -2.55 (-10.51,5.41) | -0.41 (-6.87,6.04) | -0.02 (-6.26,6.23) | CAE |  |  |
| -6.82 (-11.81,-1.84) | -3.93 (-10.62,2.76) | -1.79 (-6.59,3.01) | -1.39 (-5.90,3.12) | -1.38 (-8.35,5.60) | RE |  |
| -8.45 (-11.59,-5.31) | -5.55 (-11.00,-0.10) | -3.41 (-6.24,-0.58) | -3.02 (-5.32,-0.71) | -3.00 (-8.80,2.80) | -1.62 (-5.50,2.25) | Con |

Note:Estimates are presented as mean differences (MD) and 95% confidence intervals (CI). Table cell units are color-coded for ease of interpretation. Exercise interventions are listed in yellow text. Statistically significant results (with non-zero 95% CI) are highlighted in green.

**Table S5: Effects of Different Exercise Therapies on SDS**

| CAE |  |  |
| --- | --- | --- |
| -7.10 (-16.10,1.91) | RT |  |
| -12.48 (-17.79,-7.17) | -5.38 (-12.66,1.89) | Con |

Note:Estimates are presented as mean differences (MD) and 95% confidence intervals (CI). Table cell units are color-coded for ease of interpretation. Exercise interventions are listed in yellow text. Statistically significant results (with non-zero 95% CI) are highlighted in green.

**Table S6: Effects of Different Exercise Therapies on BI**

| RT |  |  |  |  |  |
| --- | --- | --- | --- | --- | --- |
| 1.88 (-16.00,19.76) | WQX |  |  |  |  |
| 2.24 (-9.71,14.20) | 0.36 (-18.67,19.40) | CAE |  |  |  |
| 4.26 (-6.92,15.44) | 2.38 (-16.18,20.94) | 2.02 (-10.93,14.96) | TC |  |  |
| 6.23 (-11.78,24.24) | 4.35 (-18.97,27.67) | 3.99 (-15.17,23.14) | 1.97 (-16.71,20.65) | BDJ |  |
| 9.47 (2.39,16.55) | 7.59 (-8.83,24.01) | 7.23 (-2.40,16.86) | 5.21 (-3.44,13.86) | 3.24 (-13.32,19.80) | Con |

Note:Estimates are presented as mean differences (MD) and 95% confidence intervals (CI). Table cell units are color-coded for ease of interpretation. Exercise interventions are listed in yellow text. Statistically significant results (with non-zero 95% CI) are highlighted in green.

**Table S7: Effects of Different Exercise Therapies on FMA**

| RE |  |  |  |  |  |  |
| --- | --- | --- | --- | --- | --- | --- |
| 4.90 (-4.29,14.09) | RT |  |  |  |  |  |
| 9.39 (2.86,15.92) | 4.49 (-4.16,13.14) | WQX |  |  |  |  |
| 10.05 (-1.38,21.48) | 5.15 (-7.60,17.90) | 0.66 (-10.33,11.65) | CAE |  |  |  |
| 11.60 (5.66,17.54) | 6.70 (-1.51,14.91) | 2.21 (-2.84,7.26) | 1.55 (-9.10,12.20) | BDJ |  |  |
| 12.63 (6.69,18.56) | 7.73 (-0.48,15.93) | 3.24 (-1.81,8.28) | 2.58 (-8.07,13.22) | 1.03 (-3.22,5.28) | TC |  |
| 17.55 (12.43,22.67) | 12.65 (5.01,20.29) | 8.16 (4.10,12.22) | 7.50 (-2.72,17.72) | 5.95 (2.94,8.96) | 4.92 (1.93,7.92) | Con |

Note:Estimates are presented as mean differences (MD) and 95% confidence intervals (CI). Table cell units are color-coded for ease of interpretation. Exercise interventions are listed in yellow text. Statistically significant results (with non-zero 95% CI) are highlighted in green.

**Table S8: Effects of Different Exercise Therapies on BBS**

| BDJ |  |  |  |  |  |  |
| --- | --- | --- | --- | --- | --- | --- |
| 2.57 (-5.84,10.97) | YG |  |  |  |  |  |
| 5.10 (-5.23,15.44) | 2.53 (-8.30,13.37) | RE |  |  |  |  |
| 5.59 (-2.18,13.35) | 3.02 (-5.41,11.45) | 0.49 (-9.87,10.84) | RT |  |  |  |
| 5.85 (-2.41,14.11) | 3.28 (-5.60,12.17) | 0.75 (-9.98,11.48) | 0.27 (-8.02,8.56) | TC |  |  |
| 7.50 (-2.01,17.02) | 4.93 (-5.13,14.99) | 2.40 (-9.32,14.12) | 1.91 (-7.63,11.45) | 1.65 (-8.30,11.60) | CAE |  |
| 8.20 (2.73,13.67) | 5.63 (-0.74,12.00) | 3.10 (-5.67,11.87) | 2.61 (-2.90,8.13) | 2.35 (-3.84,8.54) | 0.70 (-7.29,8.69) | Con |

**Table S9: Cochrane Risk of Bias 2.0 (RoB 2.0)**

| Author | Bias arising from the randomization process | Bias due to deviations from intended interventions | Bias due to missing outcome data | Bias in measurement of the outcome | Bias in selection of the reported result | Total |
| --- | --- | --- | --- | --- | --- | --- |
| Chen Changxiang 2013 | High risk | High risk | Some concerns | High risk | Some concerns | High risk |
| Guo Jing 2024 | High risk | High risk | Some concerns | High risk | Some concerns | High risk |
| Guo Jing 2025 | Some concerns | High risk | Some concerns | High risk | Some concerns | High risk |
| Shen Yi 2006 | Some concerns | High risk | Some concerns | High risk | Some concerns | High risk |
| Xu Hua 2023 | Some concerns | High risk | Some concerns | High risk | Some concerns | High risk |
| TorIvar Gjellesvik 2021 | Low risk | Some concerns | Low risk | Low risk | Low risk | Some concerns |
| Frederike A. Straeten 2023 | Low risk | Some concerns | Low risk | Some concerns | Low risk | Some concerns |
| PingPing Sun 2022 | Low risk | Some concerns | Low risk | Some concerns | Some concerns | Some concerns |
| Li Xiaohui 2018 | Some concerns | High risk | Some concerns | High risk | Some concerns | High risk |
| Li Yuling 2012 | Some concerns | High risk | High risk | High risk | Some concerns | High risk |
| Wang Jian 2024 | Some concerns | High risk | Some concerns | High risk | Some concerns | High risk |
| Wang Lin 2012 | Some concerns | High risk | Some concerns | Some concerns | Some concerns | High risk |
| Zhao Bin 2017 | Some concerns | High risk | Some concerns | High risk | Some concerns | High risk |
| Jie Zhao 2022 | Low risk | Low risk | Low risk | Low risk | Low risk | Low risk |
| Rhayun Song 2021 | Some concerns | Some concerns | Low risk | Low risk | Low risk | Some concerns |
| Yihan 2024 | Low risk | Some concerns | Low risk | Low risk | Some concerns | Some concerns |
| Liu Xiaoyu 2021 | Some concerns | High risk | Some concerns | High risk | Some concerns | High risk |
| Tang Zhisheng 2023 | Some concerns | High risk | Some concerns | High risk | Some concerns | High risk |
| Tang Zhisheng 2024 | Some concerns | High risk | Some concerns | High risk | Some concerns | High risk |
| Liu Xiao 2023 | Some concerns | High risk | Some concerns | High risk | Some concerns | High risk |
| Birgit Vahlberg 2016 | Low risk | Some concerns | Some concerns | Low risk | Some concerns | Some concerns |
| Taisheng Feng 2024 | Low risk | Some concerns | Low risk | Low risk | Some concerns | Some concerns |
| Wen Pengzhen 2024 | Some concerns | High risk | Some concerns | High risk | Some concerns | High risk |
| Yen 2022 | High risk | High risk | Some concerns | Some concerns | Low risk | High risk |
| Maarten A. Immink 2014 | Low risk | Some concerns | Some concerns | Low risk | Some concerns | Some concerns |
| Chen Liping 2006 | Some concerns | High risk | Some concerns | High risk | Some concerns | High risk |
| Pang Xiuping 2019 | Some concerns | High risk | Some concerns | High risk | Some concerns | High risk |
| Wang Jinlan 2018 | Some concerns | High risk | Some concerns | High risk | Some concerns | High risk |
| Yang Lizhi 2022 | Some concerns | High risk | Low risk | Some concerns | Some concerns | High risk |
| Sue-Min Lai 2006 | Low risk | Some concerns | Some concerns | Low risk | Some concerns | Some concerns |
| Mary Stuart 2009 | High risk | Some concerns | Low risk | Some concerns | Some concerns | High risk |
| Eun-Mi Jun 2012 | Some concerns | High risk | Some concerns | Some concerns | Some concerns | High risk |
| Deepak Thazhakkattu Vasu 2021 | Low risk | Some concerns | Low risk | Some concerns | Low risk | Some concerns |

# **Figures**

**Figure S1.**Forest Plot of the Effects of Different Exercise Therapies on HAMD.


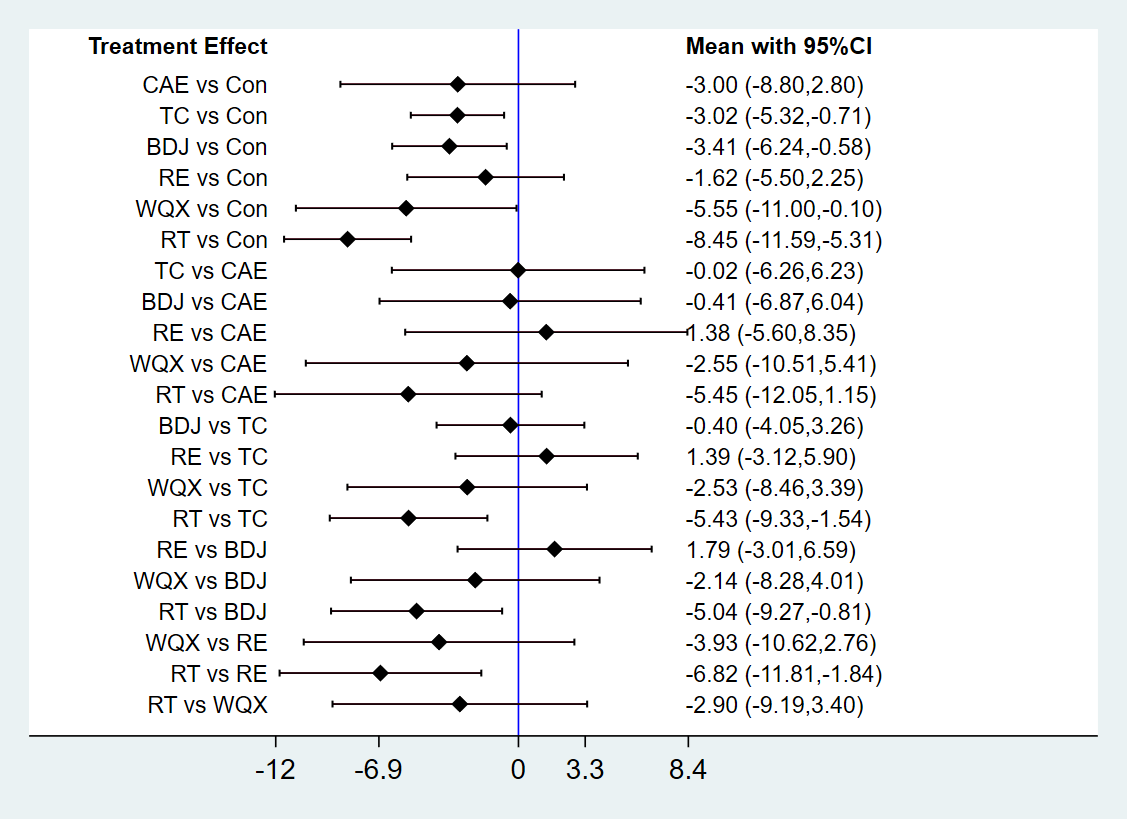


**Figure S2.**Forest Plot of the Effects of Different Exercise Therapies on SDS.


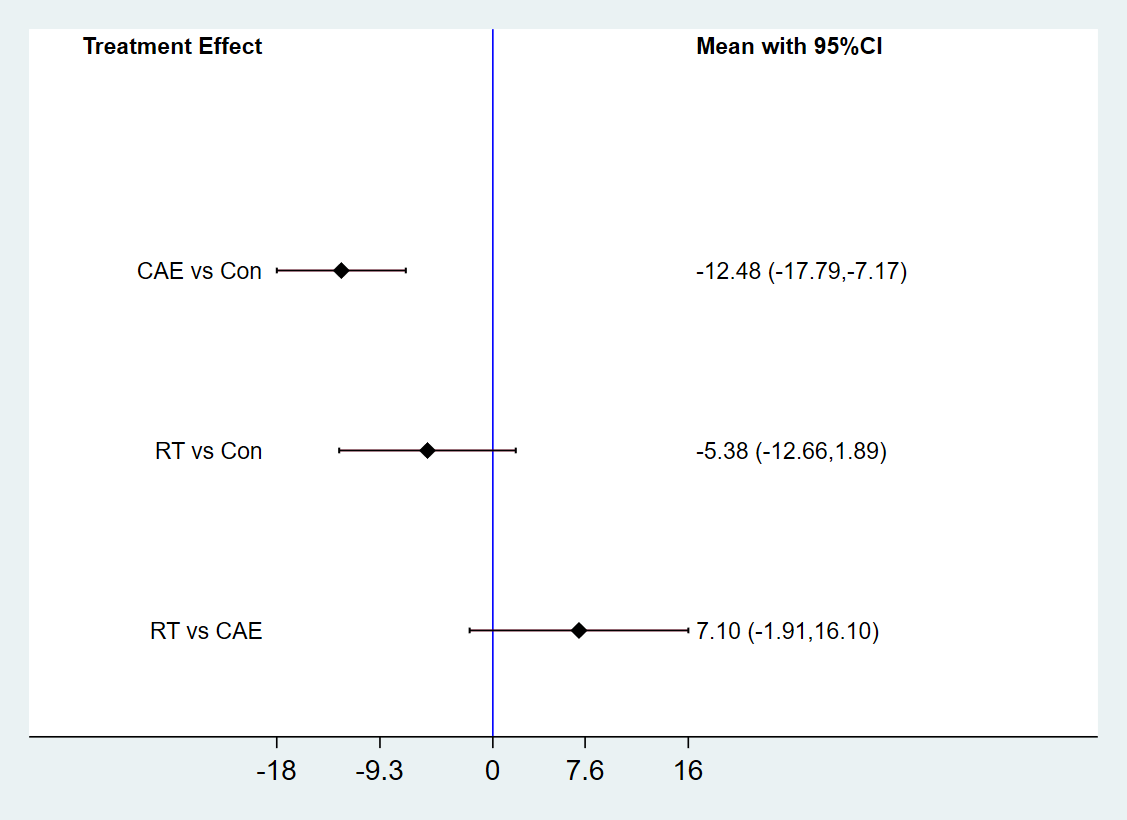


**Figure S3.**Forest Plot of the Effects of Different Exercise Therapies on BI.


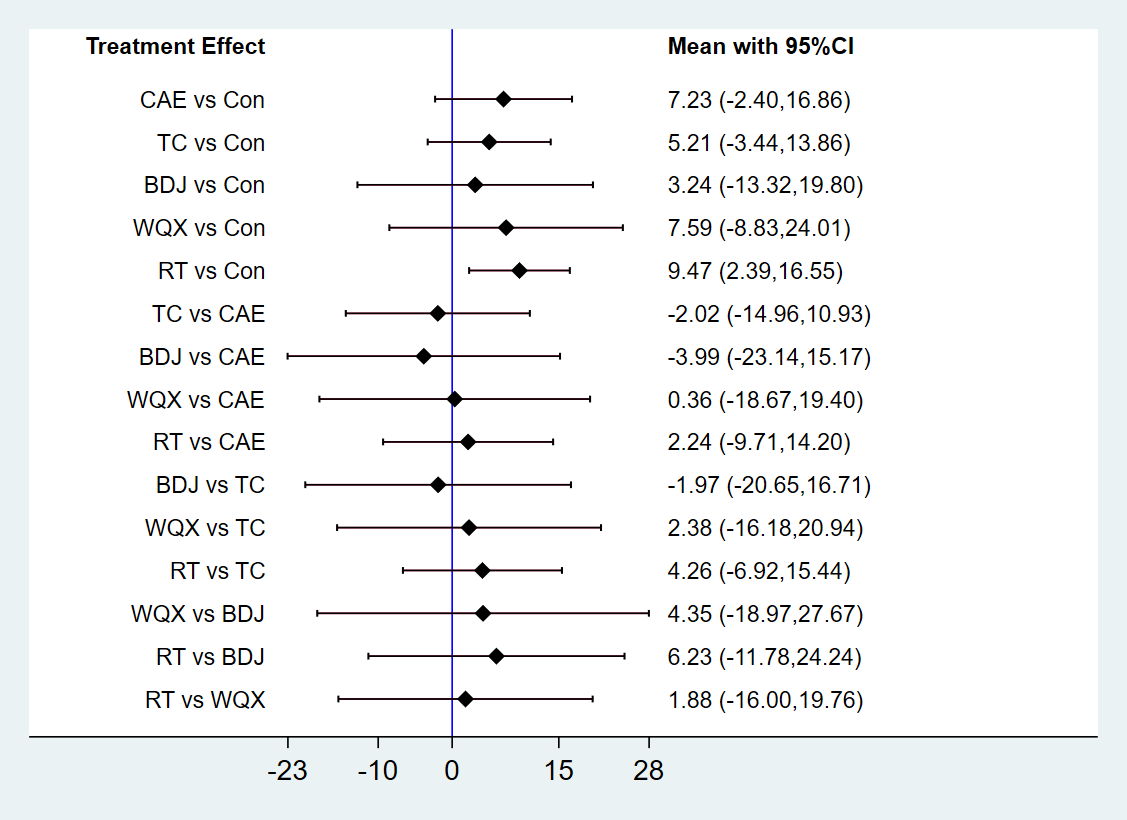


**Figure S4.**Forest Plot of the Effects of Different Exercise Therapies on FMA.


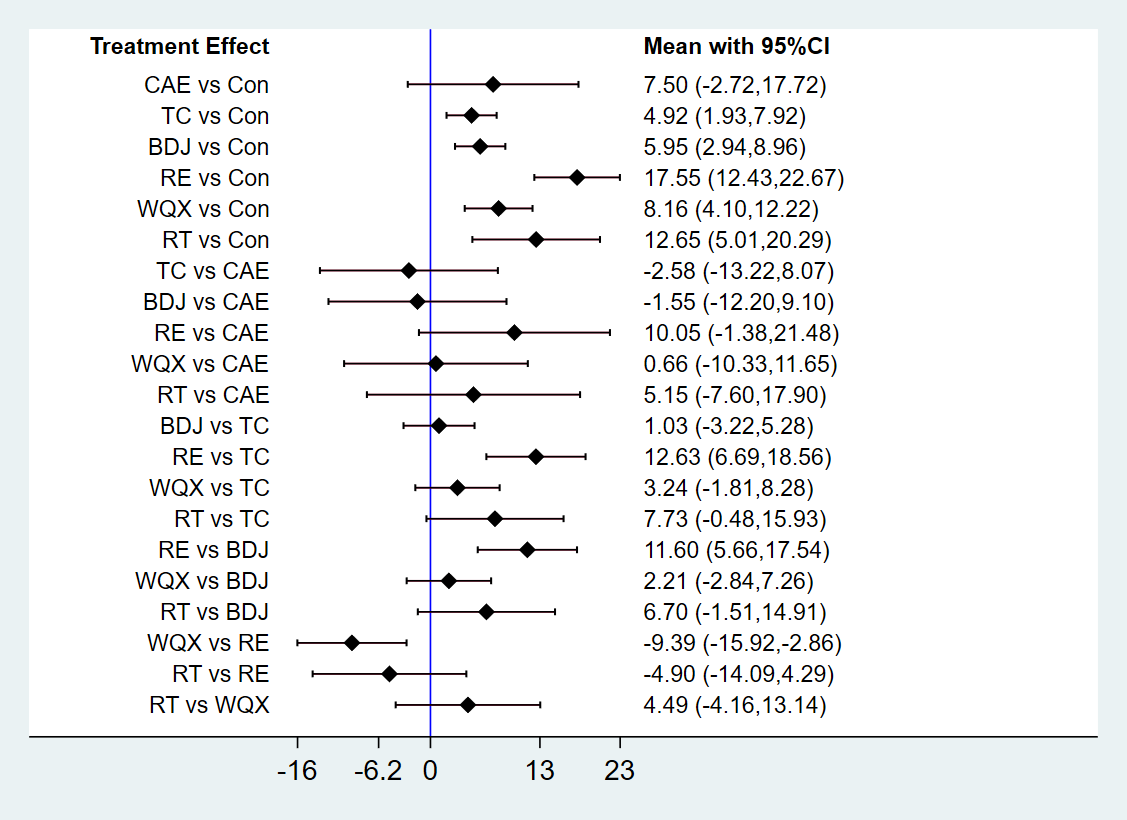


**Figure S5.**Forest Plot of the Effects of Different Exercise Therapies on BBS.


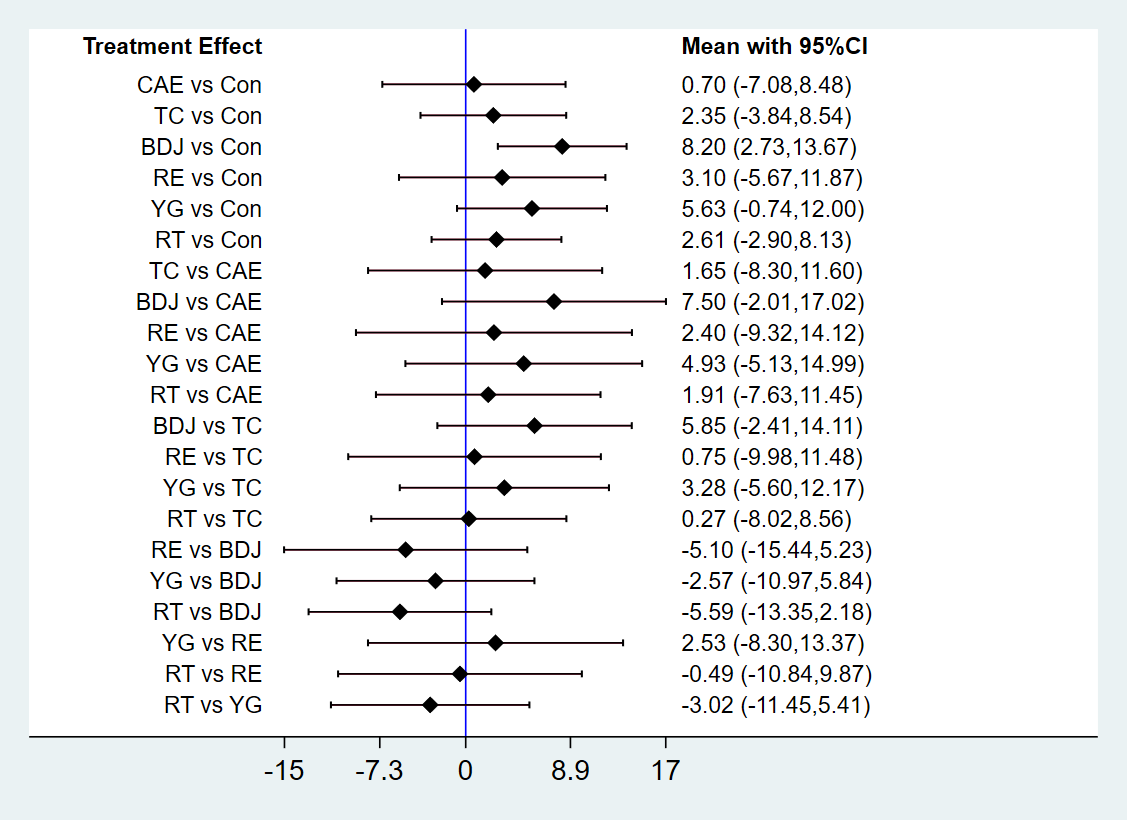

Supplement: Supplementary file 1 [file Table_1.DOCX]
